# A genome-wide DNA methylation study in colorectal carcinoma

**DOI:** 10.1186/1755-8794-4-50

**Published:** 2011-06-23

**Authors:** Muhammad G Kibriya, Maruf Raza, Farzana Jasmine, Shantanu Roy, Rachelle Paul-Brutus, Ronald Rahaman, Charlotte Dodsworth, Muhammad Rakibuz-Zaman, Mohammed Kamal, Habibul Ahsan

**Affiliations:** 1 Department of Health Studies, The University of Chicago, Chicago, IL 60637, USA; 2 Bangabandhu Sheikh Mujib Medical University (BSMMU), Dhaka 1000, Bangladesh; 3 Columbia University and University of Chicago Research Office in Bangladesh, Dhaka, Bangladesh; 4 Department of Human Genetics, The University of Chicago, Chicago, IL 60637, USA; 5 Department of Medicine, The University of Chicago, Chicago, IL 60637, USA; 6 Comprehensive Cancer Center, The University of Chicago, Chicago, IL 60637, USA

## Abstract

**Background:**

We performed a genome-wide scan of 27,578 CpG loci covering 14,475 genes to identify differentially methylated loci (DML) in colorectal carcinoma (CRC).

**Methods:**

We used Illumina's Infinium methylation assay in paired DNA samples extracted from 24 fresh frozen CRC tissues and their corresponding normal colon tissues from 24 consecutive diagnosed patients at a tertiary medical center.

**Results:**

We found a total of 627 DML in CRC covering 513 genes, of which 535 are novel DML covering 465 genes. We also validated the Illumina Infinium methylation data for top-ranking genes by non-bisulfite conversion q-PCR-based methyl profiler assay in a subset of the same samples. We also carried out integration of genome-wide copy number and expression microarray along with methylation profiling to see the functional effect of methylation. Gene Set Enrichment Analysis (GSEA) showed that among the major "gene sets" that are hypermethylated in CRC are the sets: "inhibition of adenylate cyclase activity by G-protein signaling", "Rac guanyl-nucleotide exchange factor activity", "regulation of retinoic acid receptor signaling pathway" and "estrogen receptor activity". Two-level nested cross validation showed that DML-based predictive models may offer reasonable sensitivity (around 89%), specificity (around 95%), positive predictive value (around 95%) and negative predictive value (around 89%), suggesting that these markers may have potential clinical application.

**Conclusion:**

Our genome-wide methylation study in CRC clearly supports most of the previous findings; additionally we found a large number of novel DML in CRC tissue. If confirmed in future studies, these findings may lead to identification of genomic markers for potential clinical application.

## Background

Colorectal carcinoma (CRC) is one of the most common human malignancies worldwide, and an increasing incidence of CRC in Asia has been reported [1,2]. CRC cells develop several genetic and epigenetic alterations in cancer-related genes to achieve malignant status [3]. Promoter hypermethylation coupled with loss of heterozygosity at the same locus results in loss of gene function in many tumor cells [4]. Alterations in DNA methylation in cancer, in general, have been known for 25 years, including hypomethylation of oncogenes and hypermethylation of tumor suppressor genes [5]. Identification of specific DNA methylation markers would be helpful for understanding pathogenetic mechanisms, as well as for developing new therapeutic strategies. So far most of the studies addressing DNA methylation and cancer have followed the candidate gene approach, or addressed a handful of genes, or have used cell lines [6-8]. Recently Ang *et al. *used Illumina's GoldenGate array covering 1,505 loci and found a total of 202 loci covering 132 genes to be differentially methylated in CRC [9]. Attempts are being made to classify CRC by methylation patterns that correlate with prognosis [10-15]. A recent study suggests that there may be a significant difference in DNA methylation profiles between cancer cell lines and original tumor tissue emphasizing the need to be cautious in using cell lines as a tumor model for molecular studies of cancer [16].

To our knowledge, there is no published study from Southeast Asia addressing these molecular features in CRC to better understand the underlying pathology. There is epidemiologic evidence of a link between ethnicity, certain food habits (more red meat, less vegetables) and CRC [17]. With very few exceptions in the tribal areas, the Bangladeshi population is relatively homogenous ethnically and has a more or less similar pattern of food habit. In this study we have used Illumina's Infinium methylation assay to study the methylation status in 27,578 CpG sites covering 14,475 genes in paired CRC and surrounding healthy tissue from Bangladeshi patients with CRC to identify differentially methylated loci involved in CRC.

## Methods

### Tissue Samples

The samples were collected from surgically removed colonic specimens received by the department of Pathology, Bangabandhu Sheikh Mujib Medical University (BSMMU), Dhaka, Bangladesh during the period of December 2009 to March 2010. All samples were collected by one surgical pathology fellow (MR) from the operating room immediately after the surgical resection. We considered the consecutive 24 eligible cases with histologically confirmed diagnosis of CRC. Histopathology was done independently by two histopathologists (MK & MR), and there was concordance in all 24 cases. For each patient, one sample was collected from the tumor mass, and another sample was taken from the resected unaffected part of the colon about 5-10 cm away from the tumor mass. Thus, from each individual we obtained a pair of tumor and normal tissues. From each site, the tissue was collected as fresh frozen and also in RNA-stabilizing buffer. The samples were shipped on dry ice to the molecular genomics lab at The University of Chicago for subsequent DNA extraction and methylation assay. Patient characteristics are shown in Additional File 1 Table S1. For each patient, we also abstracted key demographic and clinical data and tumor characteristics from hospital medical records. Written informed consent was obtained from all participants. The research protocol was approved by the "Ethical Review Committee, Bangabandhu Sheikh Mujib Medical University", Dhaka, Bangladesh (BSMMU/2010/10096) and by the "Biological Sciences Division, University of Chicago Hospital Institutional Review Board", Chicago, IL, USA (10-264-E).

### DNA extraction and quality control

DNA was extracted from fresh frozen tissue using Puregene Core kit (Qiagen, Maryland, USA). The average 260/280 ratio was 1.85. Electropherogram from Agilent BioAnalyzer with Agilent DNA 12000 chips showed the fragment size to be >10000 bp (Additional File 2 Figure S1).

### RNA extraction and quality control

RNA was extracted from RNA Later preserved colonic tissue using Ribopure tissue kit (Ambion, USA, Cat# AM1924). Quality was checked on Agilent BioAnalyzer. RNA from two patients showed poor quality and that was also reflected on the microarray data.

### Genome-wide methylation assay

The Infinium Methylation-27Assay was used to detect 27,578 CpG sites genome-wide, spanning 14,495 genes. The CpG sites were located within the proximal promoter regions of genes, with the distance to transcription start site (TSS) ranging from 0 to 1499 bp averaged at 389 ± 341 bp. For bisulfite conversion, EZ DNA methylation kit (Zymo Research, USA) was used. Paired samples (CRC and corresponding normal) were processed on the same chip, and all the 48 samples were processed on 4 chips (12 samples/chip) at the same time to avoid batch effect. The Illumina protocol was followed for the methylation assay. A Tecan Evo robot was used for automated sample processing and the chips were scanned on a single BeadArray reader (S-428). Control panel in the BeadStudio analytical software showed excellent intensity for staining (above 15,000), clear clustering for the hybridization probes, good target removal intensity (<400) and satisfactory bisulfite conversion.

### Validation of Infinium methylation platform by q-PCR array

We used commercially available custom Methyl profiler PCR array from Qiagen-SABiosciences, which does not require bisulfite conversion. Manufacturer's protocol (http://www.sabiosciences.com/dna_methylation_custom_PCRarray.php) was followed for the assay and ABI7900 RT-PCR instrument was used to read the plates. Basically, the assay relies on differential cleavage of target sequences by two different restriction endonucleases - methyl specific (MS) and methyl dependent (MD), whose activity require either the presence or absence of methylated cytosines respectively in their recognition sequences. Details of the assay are published elsewhere [18]. Using the standard ΔΔCt method the proportion of hypermethylated and intermediately methylated DNA was calculated [18] using the manufacturer supplied Excel macro spreadsheet.

### High density SNP assay

We used Illumina 610 Quad BeadChip (Illumina Inc.) to obtain the copy number data from a total of 620,901markers (592,532 SNPs and 28,369 CNV probes).

### Genome-wide gene expression microarray

We used HT12 v4 BeadChip (Illumina Inc.) for gene expression. The chip contains a total of 47,231 probes covering 31,335 genes. Paired samples were processed in same chip (12 samples/chip) and all 48 samples were processed in a single batch using 4 chips to minimize batch effect.

### Statistical analysis

To compare the continuous variables (e.g. number of detected loci/samples or average signal intensity/average β value etc. among the two groups), we used one-way analysis of variance (ANOVA).

### Genome-wide Methylation data analysis

For measuring methylation, we used the Illumina BeadStudio software to generate the β value for each locus from the intensity of methylated and unmethylated probes. We used the intensity values with and without background normalization. The background value is derived by averaging the signals of built-in negative control bead types, which are designed to be thermodynamically equivalent to the regular probes but lack a specific target in the transcriptome. The β is calculated as (intensity of methylated probe)/(intensity of methylated probe + intensity of unmethylated probe). Hence, β ranges between 0 (least methylated) and 1 (most methylated) and is proportional to the degree of methylated state of any particular loci. The methylation module of BeadStudio was used for differential methylation analysis using Illumina custom model. The model operates under the assumption that the methylation value β is normally distributed among biological replicates corresponding to a set of biological conditions (tumor and normal in the present scenario). DiffScore of a probe is computed as:

In addition to the Illumina BeadStudio differential methylation analysis, we exported the BeadStudio generated β-values to PARTEK Genomic Suite [19] for further statistical analyses. For statistical analysis we used these β-values with and without quantile normalization. In this way, initially we examined four sets of data - (1) no normalization of signal intensity, no normalization of β-values; (2) no normalization of signal intensity to calculate β-values initially, but subsequently quantile normalization of β-values were used; (3) background normalization of signal intensity to calculate β-values, but no normalization of β-values were used; (4) background normalization of signal intensity to calculate β-values, and quantile normalization of β-values were used. Principal component analysis (PCA) and sample histograms were checked as a part of quality control analyses of the data. Mixed-model multi-way ANOVA (which allows more than one ANOVA factor to be entered in each model) was used to compare the individual CpG loci methylation data across different groups. In general, "tissue" (tumor/adjacent normal), sex (male/female) and tumor location (proximal colon/distal colon) were used as categorical variables with fixed effect since the levels "tumor/normal", "male/female", and "proximal/distal" represent all conditions of interest; whereas "case ID#" (as proxy of inter-person variation) was treated as categorical variable with random effect, since the person ID is only a random sample of all the levels of that factor. Method of moments estimation was used to obtain estimates of variance components for mixed models [20]. As per the study design, we processed both the CRC tissue and the corresponding adjacent normal sample from one individual in a single chip (one chip accommodates 12 samples) and all the four chips required to run a total of 48 samples were run in a single batch to avoid batch effect. In the ANOVA model, the β-value for the CpG loci was used as the response variable, and "tissue" (tumor or normal), case ID#, "sex" and "location" were entered as ANOVA factors. It may be noted that "sex" and "location" were nested within "case ID#". One example of a model is as follows:

where Y_ijklm _represents the m-th observation on the i-th Tissue j-th Sex k-th Location l-th CaseID, μ is the common effect for the whole experiment, ε_ijklm _represents the random error present in the m-th observation on the i-th Tissue j-th Sex k-th Location l-th CaseID. The errors ε_ijklm _are assumed to be normally and independently distributed with mean 0 and standard deviation δ for all measurements.

In GO Enrichment analysis, we tested if the genes found to be differentially methylated fell into a Gene Ontology category more often than expected by chance. We used chi-square test to compare "number of significant genes from a given category/total number of significant genes" vs. "number of genes on chip in that category/total number of genes on the microarray chip". Negative log of the p-value for this test was used as the enrichment score. Therefore, a GO group with a high enrichment score represents a lead functional group. The enrichment scores were analyzed in a hierarchical visualization and in tabular form.

In addition to looking at differential methylation at the level of individual CpG loci, we also examined the differential methylation of "gene sets" using the Gene Set Enrichment Analysis (GSEA) [21]. Given an *a priori *defined set of genes S (sharing the same GO category), the goal of GSEA was to determine whether the members of S were randomly distributed throughout the ranked list or primarily found at the top or bottom. Considering the fact that GSEA can look at single variable (unadjusted β-value), we also used GO-ANOVA which offers adjustments for other factors such as "person-to-person" variation, "tissue type" variation etc.

GO-ANOVA is a mixed model ANOVA to test the methylation of a set of genes (sharing the same GO category) instead of an individual gene in different groups [19]. The analysis is performed at the gene level, but the result is expressed at the level of the GO-category by averaging the member genes' results. The equation for the model was:

where Y represents the methylation status of a GO-category, μ is the common effect or average methylation of the GO-category, T is the tissue-to-tissue (tumor/healthy) effect, P is the patient-to-patient effect, G is the gene-to-gene effect (differential methylation of genes within the GO-category independent of tissue types), S(T*P) is the sample-to-sample effect (this is a random effect, and nested in tissue and patient) and ε represents the random error.

### Cross-validation

For the one-level cross validation, the data was first divided into 10 random partitions. In each iteration, 10% of samples were held out for testing while the remaining 90% samples were used to fit the parameters of the model. We also used a 6 × 10 two-level nested cross-validation [22]. In the outer cross-validation, with random 1/6-th of the samples (n = 8) were held out as test samples, and the remaining 40 samples were used in an inner 10-fold cross-validation (1/10-th of these samples (n = 4) were held out at each iteration, and it was repeated 10 times) to determine the optimal predictor variables and other classifier parameters. The model that performed the best on the inner cross-validation was applied to the 8 test samples that were held-out in the outer cross-validation. This was repeated 6 times. Thus we had a total of 10 × 6 or 60 permutations for inner cross-validation and 6 for the outer cross-validation. The inner cross-validation was performed in order to select predictor variables and optimal model parameters, and the outer cross-validation was used to produce overall accuracy estimates for the classifier. Initially we tested several classification methods: (a) K-Nearest Neighbor (KNN) with Euclidean distance measure and 1-neighbor, (b) K-Nearest Neighbor (KNN) with Euclidean distance measure and 3-neighbor (c) nearest centroid with equal prior probability and (d) linear discriminent analysis with equal prior probability. Based on the results (normalized correct rate), we finally used KNN with Euclidean distance measure and 3-neighbor as the classifier, and regarding the number of variables, we tested 1 through 50 variables. For automated variable selection, we used 2-way ANOVA where tissue type and case ID# were used as ANOVA factors. One of the loci with maximum delta-β (*DAB2IP*) was forced into the model.

### Genome-wide Copy number (CN) analysis

BeadStudio normalized intensity values were imported into PARTEK genomic suit [19]. Intensity data from the normal tissue was used as reference for generating copy number data for each marker. Standard PCA and sample histogram were generated as part of QC. Genomic segmentation was done with a setting of minimum of 10 markers, p-value threshold of 0.001 for two neighboring regions having significantly differing means. A segment was considered as amplification if the mean CN was ≥2.5 and deletion if the mean was ≤1.5. The phenotype (CRC or normal) was tested for association with amplification/deletion status of the sample using Pearson's Chi-square test.

### Genome-wide Gene Expression analysis

In BeadStudio, quantile normalization was used for the intensity data. PCA detected the arrays from the same two patients as outlier which showed poor RNA quality on Agilent BioAnalyzer. Microarray data from those two patients were excluded from the analysis. Differential gene expression analysis was done using the same mixed model multi-way ANOVA [19] as in case of methylation analysis. We report genes to be differentially expressed only if that shows at least 1.3 fold change in either direction at FDR 0.05. We used this 1.3 fold as cut-off based on the power calculation from our data. Given the sample size, we had 80% power to detect 90% of the truly differentially genes at 1.3 fold.

### Correlation between methylation/gene expression and methylation/copy number

To investigate the effect of DNA methylation on gene expression, we used Spearman's rank to test correlation between the beta-value of a methylation locus and the log_2_-transformed normalized expression value of a gene within a maximum distance of 2 Kb from that methylation locus. Correlated methylation and expression data were taken from the same samples. In the same way, to see the effect of copy number on gene expression, we also used Spearman's rank to test correlation between the average copy number of a genomic segment and the log_2_-transformed normalized expression value of a gene overlapping with that genomic segment. Copy number and methylation data were also from the same samples.

## Results

Our study was conducted on 24 patients (17 male, 7 female) with CRC with a mean age of 45.5 years (SD 16.8) (Additional File 1 Table S1). There were a total of 27,578 loci covering 14,495 genes (average 1.9 CpG loci per gene) that were studied for methylation status per sample. On average, about 27,511 loci (95% CI 27,488 - 27,534) were detected in each sample at p < 0.05 level. A locus was said to be detected at p < 0.05 level if the mean signal intensity from multiple probes for that CpG locus was significantly higher (at the level of p < 0.05) than the negative control on the same chip. Mean number of loci detected at p < 0.05 in tumor and normal tissue was similar: 27483.88 (SD 114.75) vs. 27507.25 (SD 65.84) (p = 0.41). There was a very strong correlation (r^2 ^= 0.9932) of the total signal intensity (methylated and unmethylated) of the 27,578 loci between the 24 normal tissues and corresponding 24 tumor tissues suggesting uniform amplification and hybridization for all samples. However, when the average β of tumor tissue samples were plotted against that of corresponding normal tissue samples, there were clearly a number of loci that were differentially methylated in CRC tissues compared to normal tissues. The data discussed in the publication have been deposited in NCBI's Gene Expression Omnibus [23] and will be accessible through GEO Series accession number GSE29490 (http://www.ncbi.nlm.nih.gov/geo/query/acc.cgi?acc=GSE29490).

### Sources of variation in the methylation data

**Figure 1:**
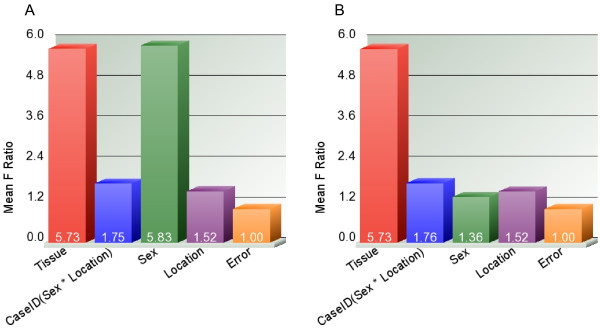
**A and B Sources of variation in methylation data**. Statistical significance of the different sources of variation in the methylation data estimated by 4-way ANOVA models. F-ratio for each factor (source) represents the F-statistics for that factor/F-statistics for error (noise). 1A shows the result in all 27,578 loci and 1B shows similar result only in the autosomal loci (n = 26,486) depicting the effect of sex chromosomal markers.

Principal component analysis (PCA) suggested a clustering of samples by tissue type (not shown). In the next step, to further investigate the source of variation in the expression, we used multivariate ANOVA. Figure 1A shows the significance of different sources of variation in the entire data in ANOVA model where tissue type (tumor/normal), person-to-person variation (case ID#), sex and location were entered as explanatory variables at a time for the β-value (representing methylation status). The figure shows that "tissue type" and sex were the most significant sources of variation.

### Differential methylation of colon tissue in male and female subjects

When we compared all 27,578 loci in females with those in males, there were a total of 568 loci differentially methylated at FDR 0.01 level. Interestingly, 551 of them (97.0%) were in the X-chromosome, 2 were in the Y-chromosome and 15 were distributed in the autosomes. This finding is explained by the X-inactivation process, in which one of the two copies of genes on the X chromosome in females is silenced. Considering this fact, we excluded all the sex chromosome markers (n = 1092, of which 1085 in X-chromosome and 7 in Y-chromosome) from subsequent analysis for differential methylation in CRC compared to normal. The significance of different sources of variation in the methylation data in autosomes only is shown in Figure 1B.

### Differential methylation in colorectal carcinoma tissue compared to adjacent normal colon tissue

In a total set of 48 samples (tumor and corresponding adjacent normal tissue from 24 patients with CRC), we looked at genome-wide differential methylation in CRC tissue compared to normal tissue. There were a total of 26,486 CpG loci in the autosomes covering 13,890 genes. Here we present the analysis of β-value calculated from background normalized signal intensity. No further normalization of derived β-value was used. In the methylation module of BeadStudio, using the in-built Illumina custom model, we found a total of 875 significantly differentially methylated autosomal loci in CRC tissue compared to normal colonic tissue, of which 275 were hypomethylated (DiffScore = < -30 and delta-β = <-0.2) and 600 were hypermethylated (DiffScore > = 30 and delta β> = 0.2). Univariate and unpaired analysis was used for this.

**Figure 2:**
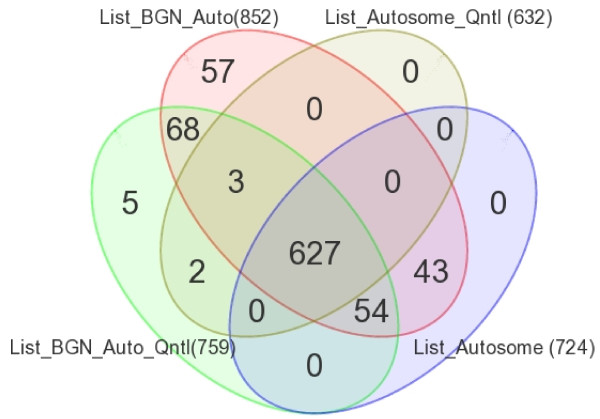
**Overlap of results from different normalization methods**. Venn diagram showing overlap between the lists of differentially methylated autosomal loci identified by ANOVA models from four different normalization procedures- (a) no normalization of signal intensity, no normalization of β-values- right lower ellipse (n = 724) (b) no normalization of signal intensity to calculate β-values initially, but subsequently quantile normalization of β-values- right upper ellipse (n = 632); (c) background normalization of signal intensity to calculate β-values, but no normalization of β-values- left upper ellipse (n = 852) and (d) background normalization of signal intensity to calculate β-values, and quantile normalization of β-values- left lower ellipse (n = 759).

In the next step, we used multi-way mixed model ANOVA to identify differentially methylated loci in CRC after adjustment for sex, "person to person variation" and location of the tumor (proximal colon or distal colon). To be conservative, we report only differentially methylated loci with absolute delta β of at least 0.2 at FDR 0.01. Following this criteria, we found a total of 852 differentially methylated loci covering 691 genes (see the List_BGN_Auto in the Venn diagram, Figure 2). Figure 2 also shows the lists of differentially methylated loci found using the same criteria (absolute delta β 0.2 at FDR 0.01) for the data with different normalization procedures (as described in statistical method section). There was a good overlap between the normalization procedures, and 627 loci were common to all the analyses. In other words, irrespective of normalization methods (with or without background normalization for signal intensity, with or without quantile normalization of the calculated β-value) these 627 loci were differentially methylated in CRC tissue compared to corresponding normal colonic tissue even after adjustment for sex, person-to-person variation and location of the tumor. Unsupervised clustering based on the common differentially methylated loci divided the samples into two main clusters, and most of the CRC samples were clustered together (figure not shown).

**Figure 3:**
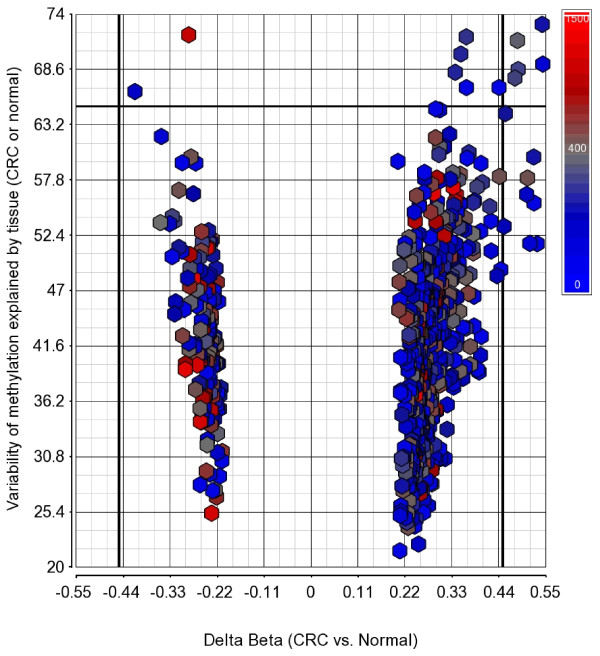
**Volcano plot of 627 significantly differentially methylated loci**. For the 627 DML, the magnitude of differential methylation (Delta β) is shown in the x-axis and the variability of β value explained by the tissue type (CRC or normal) is shown on the y-axis. Color coding was done by the distance of the locus from the transcription start site (TSS), where blue indicates close & red indicates away from the TSS.

**Figure 4:**
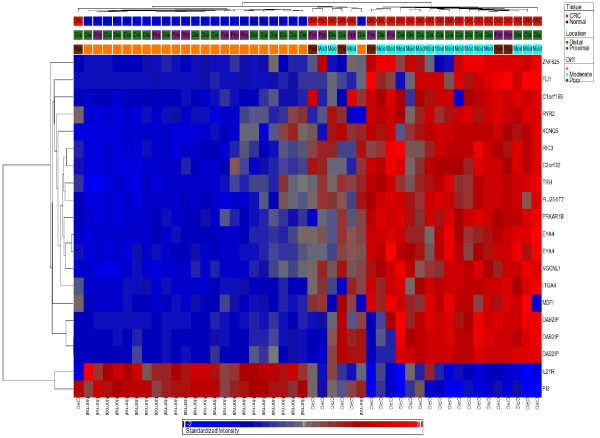
**Heatmap of 20 highly differentially methylated loci**. Unsupervised hierarchical clustering of 20 DML (rows) in 48 samples (columns) that are shown in Figure 3. These 20 DML represent the most highly differentially methylated loci (Δ β ≤ -0.45 or ≥ 0.45 with FDR 0.01) and/or whose variation could be explained by tissue type (at least 65%) in our ANOVA models. Location of the tumor (proximal or distal colon) and the differentiation of the tumor (moderately differentiated or poorly differentiated) are shown above the heatmap.

**Figure 5:**
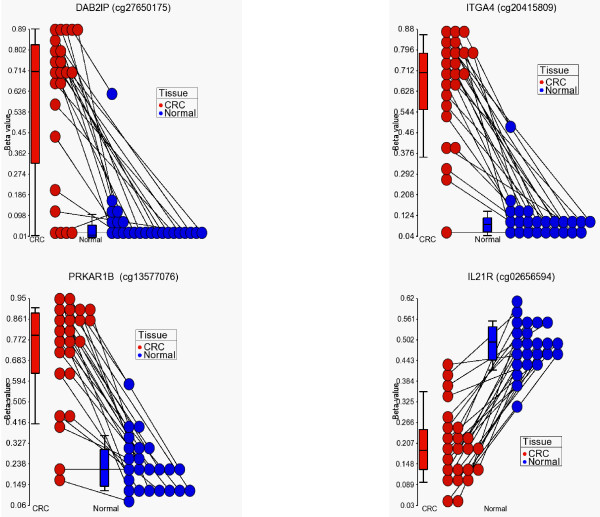
**Dotplot showing differential methylation**. The β-values for four genes (*DAB2I, ITGA4, PRKAR1B *and *IL21R*) in both normal (blue) and CRC (red) tissue samples. Connecting lines indicate paired samples (indicating same patient). *DAB2I, ITGA4 *and *PRKAR1B *show hypermethylation in the CRC samples when compared to the normal tissue and *IL21R *shows hypomethylation in the CRC samples when compared to the normal tissue.

For further analysis, we focused on these 627 common differentially methylated loci, of which 479 CpG loci were hypermethylated (median distance from TSS 219 bp) and 148 were hypomethylated (median distance from TSS 308 bp) in CRC. Hypomethylated loci were slightly more distal from TSS (Mann-Whitney U test, p < 0.001). On average, 42.8% of the variation in the methylation status of these 627 CpG loci could be explained by tissue (tumor or normal), 26.6% of the variation could be explained by person-to-person variation, 4.52% of the variation was due to sex, 1.42% was due to location and 24.66% of the variation could not be explained by the ANOVA model. Within this list of 627 loci, if we look at the greater magnitude of differential methylation (Delta β = <-0.45 or > = 0.45) or the variation of that loci explained by the tissue type (at least 65%), there were 20 loci covering 17 genes (see Figures 3 and 4). The hypermethylated genes include *FLJ25477, ITGA4, DAB2IP, KCNQ5, ZNF625, C1orf165, PRKAR1B, MDFI, C2orf32, RYR2, FLI1, RIC3, TRH, VGCNL1, EYA4 *(for q-PCR validation we selected the genes from this list) and the hypomethylated genes include *IL21R *and *PI3*. It may be noted that *DAB2IP *is a known tumor suppressor gene that has been reported to be associated with other cancers [9,24,25]; *ITGA4 *is also reported to be required for lymphangiogenesis & tumor metastasis [26]. The β-values of some of these loci in CRC and corresponding normal tissue are shown in Figure 5.

In fact, there was also a very good overlap between the univariate analysis of BeadStudio Methylation module and the mixed model multi way ANOVA analysis. A total of 832 loci were common between the lists generated by univariate Illumina custom model (n = 875) and the list generated by mixed model multi way ANOVA analysis (n = 852) as mentioned above.

If we looked at the multiple loci near a single gene, usually all the loci showed a similar trend. For example, there were six loci for the gene *ESR1*, and all were hypermethylated in CRC. We also took the average β values of multiple markers from the same gene and looked for differentially methylated genes in CRC (not shown here), and the result was almost similar to what we see with probe level analysis.

### GO Enrichment Analyses of the lists of differentially methylated genes in colorectal carcinoma tissue compared to adjacent normal colon tissue

**Figure 6:**
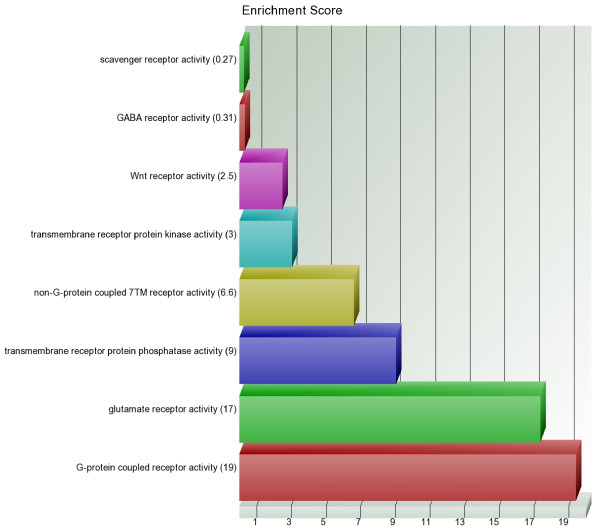
**GO-Enrichment Analysis of the list of hypermethylated loci**. Enrichment score (ES) of subgroups under "Transmembrane receptor activity" which was the most enriched (ES = 30) among the "molecular function" category.

We examined the list of 627 loci covering 513 genes (479 loci representing 374 genes were hypermethylated and 148 loci representing 139 genes were hypomethylated) to see if any particular group of genes were found to be differentially expressed in the ANOVA models more frequently than by chance. The Gene Ontology (GO) database (http://www.geneontology.org) categorizes genes on the basis of (a) "molecular function", (b) "biological process" and (c) "cellular component". For example, the number of hypermethylated loci (n = 479) in the list represent only 1.8% of the total autosomal loci (n = 26486). In other words, if 479 loci were picked randomly, then we would not expect more than 1.8% of the loci from any particular category to be present in that list. GO-enrichment analysis tests if a group of genes is overrepresented (i.e., enriched) in a list than would occur by chance. The higher the Enrichment Score (ES), the more significant the enrichment is. This is used for ranking the groups. For the list of hypermethylated loci, if we use "molecular function" for categorization, then we see that the top-ranking groups of genes that were enriched include "transmembrane receptor activity", "receptor activity", "transcription factor activity" "G-protein coupled receptor activity", "ionotropic glutamate receptor activity", "glutamate receptor activity", "extracellular-glutamate-gated ion channel activity" and "transmembrane receptor protein phosphatase activity". In other words, most of these groups or subgroups are under the broader categories of "receptor activity" or "transcription factor activity" (Figure 6). Similarly, if we use "biological process" for categorization, then the genes under the broader category "cellular developmental process" and its sub-class "cell differentiation" were highly enriched in the list of hypermethylated loci.

### Differential methylation profile at "gene set" level in colorectal carcinoma

**Figure 7:**
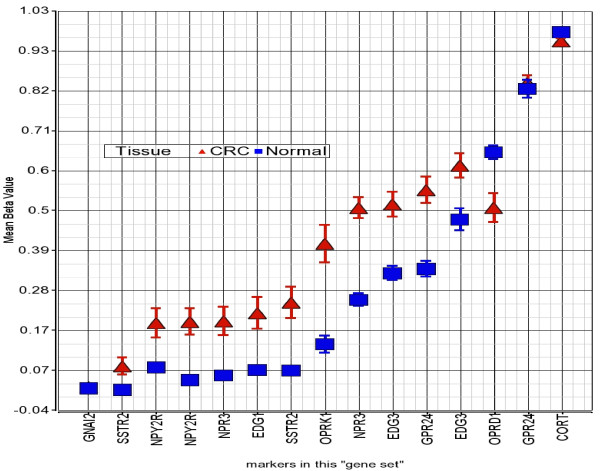
**GO-ANOVA for a "gene set"**. GO-ANOVA result for differential methylation of the gene set "inhibition of adenylate cyclase activity by G-protein signaling". The markers in this "gene set" are shown in the x-axis and their corresponding β-value is presented on y-axis. Error bars represent SE.

After looking into the differential methylation at the individual gene level, we also looked for differential methylation of different "gene sets" (different groups of genes) in colorectal carcinoma by using Gene Set Enrichment Analysis (GSEA) as well as GO-ANOVA. Gene sets were defined using publicly available data from the GO website (http://www.geneontology.org). GSEA revealed that a total of 512 "gene sets" were differentially methylated as the normalized enrichment score (NES) was = <-1.5 (n = 270, hypomethylated) or NES > = 1.5 (n = 242, hypermethylated). Using the permutation p-value for the Enrichment Score (ES) as a cut-off, a total of 220 "gene sets" were differentially methylated at p = <0.01. In GO-ANOVA analysis a total of 2851 "gene sets" crossed the threshold of FDR 0.01, and a total of 932 gene sets showed an average delta-β of = <-0.1 (n = 384, hypomethylated) or > = 0.1 (n = 548, hypermethylated). Additional File 1 Table S2 shows the results from GSEA as well as GO-ANOVA for the gene sets that had p = <0.01 for ES, NES either = <-1.5 or > = 1.5 in GSEA and also showed significant GO-ANOVA p-value at FDR 0.01 and an average delta β = <-0.1 or > = 0.1. The gene sets are arranged by NES in descending order in Additional File 1 Table S2. Obviously sorting by GO-ANOVA p-value would change the ranking. The major groups or "gene sets" that are hypermethylated in CRC are "inhibition of adenylate cyclase activity by G-protein signaling" (Figure 7), "Rac guanyl-nucleotide exchange factor activity", "regulation of retinoic acid receptor signaling pathway" and "estrogen receptor activity".

### Comparisons to colorectal carcinoma signatures from prior studies

We compared our conservative list of 513 differentially methylated genes to lists obtained by a number of previous studies. In a 2010 review paper, Kim *et al. *[27] compiled a comprehensive list of differentially methylated genes in CRC tissue and other clinical samples from patients with CRC. These were mainly candidate gene approach based studies. There were a total of 59 unique genes reported to have differential methylation in CRC tissue. Out of those 59 genes, 40 of them were also studied in our present study. In fact there were 245 loci on the chip we used that covered these 40 genes. It may be noted that 17 of those 40 genes (42.5%) were also found in our conservative list of 513 differentially methylated genes. In addition to comparing the gene lists, we also looked at the differential methylation pattern of these 245 loci covering those 40 genes in our setting. A volcano plot (Additional File 3 Figure S2A) clearly shows that most of these loci were differentially methylated, but the number actually depends on the strictness of the criteria used to define differential methylation. In fact 32 of them (80%) were significant at FDR 0.05 level without considering the delta β. Recently Ang *et al. *published a study on CRC using a genome wide approach using Illumina's GoldenGate methylation panel of 1505 CpG loci [9]. They reported a total of 202 differentially methylated loci in CRC covering 132 genes [9]. Of those 132 genes, 37 were common to our list. But again, if we look at the methylation data, then we see that in our assay there were a total of 376 loci covering those genes and 263 of those loci (70%) were also differentially methylated at FDR 0.05 in our data set (Additional File 3 Figure S2B). Therefore, it is important to take the selection criteria of a list into account while comparing the gene list. Hence, our genome-wide methylation assay not only clearly supports most of the previous findings from the literature, but in addition to that we found a large number of novel differentially methylated loci in CRC tissue compared to surrounding healthy colon tissue. The complete list of differentially methylated loci is presented as additional material (Additional File 1 Table S3) that shows the loci that have been previously reported as well as the novel loci found in present study. Kim *et al. *[27] also compiled a list of genes that were reported in the literature to be differentially methylated in other clinical samples (serum/plasma or stool) from CRC patients. The authors reported a total of 19 unique genes (there were 129 loci in the chip we used that covered 18 of these 19 genes) and interestingly 9 (50%) of those genes were also found in our conservative list of 513 differentially methylated genes in CRC. In fact, 63 of the loci covering 14 of those 18 genes (77.8%) were significantly differentially methylated at FDR 0.05 level.

In addition to looking only at CRC methylation signatures in the literature, we also compared our list to that of hypo- and hyper-methylated genes in cancer as a whole. In a 2009 review, Pogribny *et al. *compiled a list of 38 unique genes that are reported to be hypomethylated in different human cancers [28]. Only four of those (*ESR1, HSPE2, TCL1 *and *TNFRSF8*) were common in our list, and we found that all four of them were found to be hypermethylated to some degree in CRC in our study. In contrast these four genes were reported to be hypomethylated in different cancers - endometrial carcinoma, prostate cancer, T-cell Lymphocytic leukemia and in Hodgkin lymphoma respectively.

In a2007 review, Esteller *et al. *[29] compiled a list of 47 genes that were reported to be hypermethylated in different human cancers. Eight of those (*CDH13, CDKN2A, ESR1, TMEFF2, GATA4, SFRP1, TP73 *and *SOCS3*) were common in our list and all were hypermethylated in CRC in our study.

DNA repair genes are known to be important for the pathogenesis of carcinoma in general. In a review, Ronen and Glickman compiled a list of 261 DNA repair genes [30]. It was interesting to note that none of these 261 genes were common to our list of 513 genes with differential methylation in CRC.

### Uses of methylation signatures

We attempted to identify some models for differentiating CRC samples from normal samples based on methylation status. The models were identified using a 2-level nested cross validation method. Though an independent sample set was unavailable for this study, this method provided a means to estimate the accuracy of the models that may be expected in an independent set of samples. However we agree that statistical model can not replace the need of validation in an independent set of real samples.

The overall idea was to set aside a random set of samples, and then use the rest of the samples from the present study to identify an optimal combination of loci that would classify the samples as CRC or normal, and then to test the expected accuracy and different test characteristics [sensitivity, specificity, positive predictive value and negative predictive value] of the model in a different set of samples that was held out initially. Table 1 shows the summary of different models. For example, the model with 4 loci - Illumina ID# *cg02656594, cg13577076, cg20415809 *and *cg27650175 *is expected to correctly predict the diagnosis (normal or CRC) in 94% of the samples. These loci are located close to the transcription start site of the genes *IL21R, PRKAR1B, ITGA4 *and *DAB2IP *respectively. It may be noted that the other models involving more variables also give reasonable sensitivity (around 89%), specificity (around 95%), positive predictive value (around 95%) and negative predictive value (around 89%). Therefore, these markers may be considered for clinical application.

**Table 1:** Results from two-level nested cross-validation

No. of variables in KNN model	Expected test characteristics in another set of data				
	
	Accuracy	SEN (%)	SPEC (%)	PPV (%)	NPV (%)
4 variables	94.44	91.67	95.83	95.65	92.00
6 variables	92.36	87.50	95.83	95.45	88.46
8 variables	92.36	87.50	95.83	95.45	88.46
10 variables	92.36	87.50	95.83	95.45	88.46
21 variables	93.05	89.58	95.83	95.56	90.20
31 variables	92.36	87.50	95.83	95.45	88.46
41 variables	92.36	89.58	95.83	95.56	90.20
51 variables	93.06	89.58	95.83	95.56	90.20

K-Nearest Neighborhood model with Euclidean distance measure & 3 neighbor was used; SEN: sensitivity; SPEC: specificity; PPV: Positive Predictive Value; NPV: Negative Predictive Value.

We also attempted to identify methylation signatures that could differentiate histopathological findings in CRC. Although the present study was not designed to address this issue, we analyzed the 24 CRC samples using different phenotypes, including tumor stage, tumor grade, differentiation of the tumor, tumor infiltration by lymphocytes, extracellular mucin and signet ring cell. Irrespective of the histopathological diagnosis (adenocarcinoma or mucinous adenocarcinoma) and age at diagnosis (= <45 yrs vs. >45 yrs), a total of 14 loci were significantly differentially methylated in CRC of the proximal colon than those of the distal colon. Unsupervised hierarchical clustering of those loci in the CRC samples is shown in the Additional File 4 Figure S3. GO-Enrichment analysis of these 14 genes showed significant enrichment of genes related to "gut morphogenesis".

### Results from q-PCR validation

**Figure 8:**
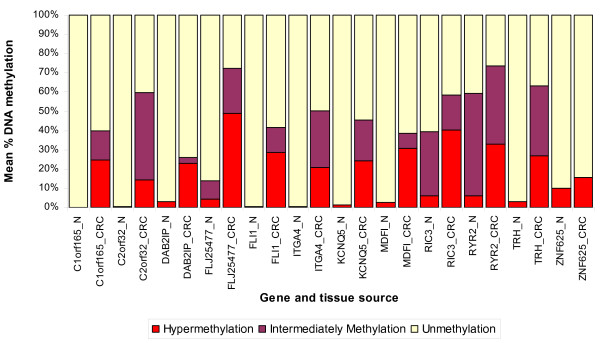
**Validation of methylation by q-PCR**. Differential methylation of selected genes in CRC tissue (_CRC) compared to adjacent normal colon mucosa (_N), as determined by Methyl Profiler q-PCR assay.

We selected the top-ranking hypermethylated 12 genes (shown in Figure 3) for validation of the Infinium methylation platform data using a methyl profiler assay. Assay development was not feasible for *PRKAR1B *for technical reasons and so that gene was replaced by the next highest-ranking gene, *TRH*. For validation we used paired DNA samples from 10 randomly selected patients from the same sample set. To compare the data from q-PCR to the β-value in the microarray, we added the proportion of intermediate methylation and hypermethylation in the q-PCR data to obtain the proportion of methylated DNA. Figure 8 and Additional File 1 Table S4 summarizes the differential methylation of these genes in CRC tissue compared to corresponding adjacent normal colonic mucosa. It may be noted that 10 out of these 12 genes were also found to be significantly hypermethylated in the q-PCR experiment. Among these 10 validated genes, 7 are novel (*TRH, C2orf32, FLJ25477, KCNQ5, C1orf165, MDFI and RIC3*) and the remaining three (*ITGA4, DAB2IP and FLI1*) were previously reported by others [9,27]. The correlation coefficients ("r") also suggest reasonably good correlation between the q-PCR data and the microarray data (Additional File 1 Table S4).

### Does the differential methylation status correlate with chromosomal abnormalities and differential gene expression in CRC?

**Figure 9:**
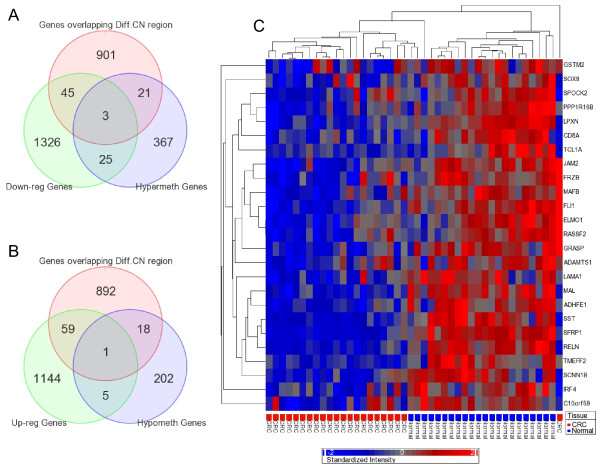
**A Overlap of hypermethylation, down-regulation and differential copy number state**. **B**: Overlap of hypomethylation, up-regulation and differential copy number state. **C**: Heatmap of differentially expressed genes: Hierarchical clustering of the gene expression data from the 25 hypermethylated genes (rows) shown in Figure 9A across the samples (columns). These genes do not overlap with regions showing significantly different copy number state in CRC tissue. The clusters generated (top dendogram) in this analysis separated most of the CRC tissues from the normal colonic mucosa tissues.

To address this issue, we also did a high density oligonucleotide SNP array (610 Quad) to detect cytogenomic abnormalities and a genome-wide gene expression assay (HT12 v4), for the same 24 patients. We detected a total of 1196 genomic segmentation regions (harboring 970 genes), for which the copy number significantly (p < 0.05, chi-square test) differs between CRC and normal mucosa. We also detected a total of 1399 down-regulated and 1209 up-regulated genes (at least 1.3 fold at FDR 0.01) in CRC compared to normal mucosa. Only 28 hypermethylated genes were down-regulated, and 48 down-regulated genes were among the genes located in genomic regions that show significant copy number change in CRC compared to normal colonic mucosa (Figure 9A). Similarly, only 6 of the hypomethylated genes were up-regulated in CRC, and 60 up-regulated genes were among the genes located in genomic regions that show significant copy number change in CRC compared to normal colonic mucosa (Figure 9B). In other words, there are relatively few genes for which either differential methylation or copy number change alone can account for the observed changes in gene expression. This clearly depicts the complexity of genomic and epi-genomic interplay in carcinogenesis. Figure 9C shows the heatmap for gene expression of those 25 genes which are down-regulated by hypermethylation irrespective of CN status.

**Figure 10:**
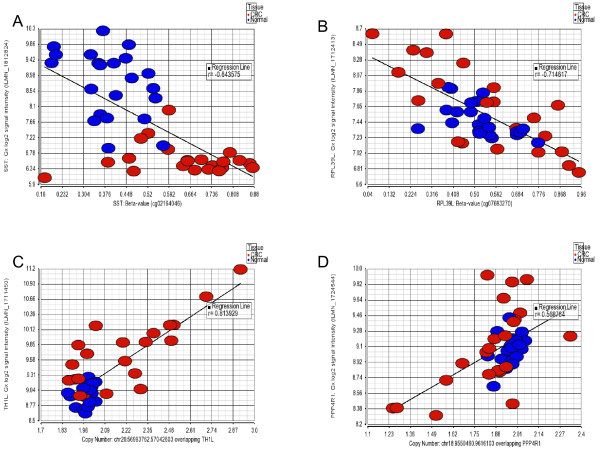
**Correlation of gene expression with methylation and copy number data**. **A and B**: Examples of gene expression (y-axis) correlating to methylation (x-axis) with differential expression & methylation in CRC compared to normal (A) and without differential expression or methylation (B). **C and D**: examples of gene expression (y-axis) correlating to copy number (x-axis) with differential expression & CN change in CRC compared to normal (C) and without differential expression or CN change (D).

In general, statistically significant *cis*-correlation (with in 2 kb region, with rank correlation p = <0.05) between methylation and gene expression was observed at 704 loci. However, only a few of these genes were differentially methylated or expressed in CRC compared to normal tissue (Figure 10A as example), while for many of the genes the methylation status correlated with gene expression at sample level without being differentially methylated or expressed in CRC tissues (Figure 10B). In the same line, we found that in a total of 3850 genomic segmentation regions, the gene expression was significantly correlated (rank correlation p = <0.05) to copy number status of the region harboring the gene. But only a few of these genes were differentially expressed or showed differential CN change in CRC compared to normal tissue (Figure 10C as example), while for many of the genes the expression level correlated with genomic CN status at sample level without being differentially expressed or having differential CN status in CRC tissues (Figure 10D as example).

## Discussion

There are only a few studies addressing the genome-wide methylation in colorectal carcinoma [9,31-33]. Very recently Kim et al [32] (Feb 2011) and Oster et al [33] (Mar 2011) used the same commercially available Infinium methylation 27 arrays in CRC and identified differentially methylated sites in Korean and European population respectively. Oster's study used carcinoma and normal tissue from different individuals for methylation analysis, whereas ours used paired tumor and adjacent normal tissue from the same patient. This allowed us to eliminate inter-individual variation in our methylation analysis, which may be one reason why our study detected a larger number of differentially methylated genes in carcinoma. Kim's study compared the methylation in paired samples like ours, but they looked at gene expression in a different set of individuals. While they looked for effect of methylation on gene expression, they could not find statistically significant difference in the mRNA expression level between promoter hypermethylation group and hypomethylation group, whereas we were able to calculate correlation coefficients using paired data in every gene and found several significant correlations. The only other genome-wide methylation study in CRC addressing promoter CpG loci using commercially available array, used much lower density array (only 1505 CpG loci) [9].

Our study in south-east Asian population suggests that, in comparison to the normal colonic mucosa, the corresponding CRC tissue shows a large number of differentially methylated loci within the CpG islands close to the transcript start site of genes, indicating the role of DNA methylation in the pathogenesis of colorectal carcinoma. The results from our study not only confirm the findings from many previous candidate gene approach based studies, but we also report a large number of novel loci that show differential methylation in CRC.

We noticed the influence of sex on genome-wide methylation that is explained by the X-inactivation process, in which one of the two copies of genes on the X-chromosome in females is silenced. A similar finding was also recently reported by Liu *et al. *[34].

Laird *et al. *[35] has recently focused on the different statistical issues for methylation data. We applied different normalization methods and found considerable overlap between the results. Use of stringent criteria for selecting differentially methylated loci and the considerable overlap between the results from different analyses, the 2-level cross validation and finally the q-PCR validation in subset suggest that we detected the truly differentially methylated loci in CRC.

Recently Irizarry *et al. *used a Comprehensive High-throughput Array for Relative Methylation (CHARM) assay to show that most methylation alterations in CRC occur up to 2000 bp away from the CpG islands themselves [31]. Because of the design of the chip used in the present study, we did not have the opportunity to look at the differential methylation at loci > 1500 bp away from the TSS. However, similar to results from Irizarry *et al. *[31], we also found that the hypomethylated loci were slightly more upstream than the hypermethylated loci.

The cross-validation results are very encouraging as a potential biomarker, but we have cross-validated only in colon tissues and not in circulating plasma DNA. In the future we would like to test the markers in an independent sample set of circulating plasma or serum DNA in CRC patients and healthy individuals. Recently He *et al. *[36] selected three methylation markers from the published literature and tested the practical use of those markers in peripheral blood sample from CRC patients. They found a sensitivity of 81% and a specificity of 90%. We had the advantage of profiling a very large number of CpG loci in paired CRC and normal colonic mucosa tissue, and our 2-level cross validation suggested that the four markers could be used as biomarkers with slightly better test characteristics.

Tanaka et al. [37] have recently applied an analytical strategy known as structural equation modeling to understanding methylation in CRC. Using a large database of over 800 samples, the authors were able to construct causality pathways of KRAS and BRAF mutations, as well as various phenotypes, on methylation of specific genes. This strategy was not feasible for our current study because of our smaller sample size and because we had not obtained information on KRAS and BRAF mutations. Nonetheless, it will be valuable for our planned future study with an expanded cohort.

Illumina's methylation assay has been compared to other platforms by others and has shown dependable results with the correlation ranging from 0.8 to 0.9 [32,33,38]. We also have validated the methylation data form Infinium methylation for 12 of the highly differentially methylated genes in our study and also found similar high correlations with Methyl Profiler assay (see Additional File 1 Table-S4 and Additional File 5 Figure S4). In another study, reproducibility tests of Infinium methylation platform was reported to have correlation greater than 0.98 between technical replicates [39]. We are aware of the fact that Illumina's Methylation27 assay detects the methylation status of on average ~2 CpG sites per gene for most genes. However, for the genes for which there were multiple CpG loci on the array (e.g. *ESR1 or DAB2IP*), we found all of the loci to be differentially methylated in the same direction. We also validated Illumina's platform in the top-ranking genes by methyl profiler PCR array which is (a) not dependent on bisulfite conversion and also (b) provides an overall methylation status of the target region as opposed to single loci. This paper was focused mainly to look at DML in CRC. However, we have also explored the link between chromosomal abnormalities (copy number), methylation and gene expression. Regulation of gene expression is complex and is not dependant only on methylation status or copy number status. Using integration of molecular cytogenetics, genome-wide copy number and expression microarray profiling, Camps et al have demonstrated the effect of copy number on gene expression in CRC [40]. To our knowledge, our study is the first one to comprehensively look at the genome-wide methylation, copy number and gene expression - all three together in primary CRC tissue. In our study, expression of a small proportion of genes was found to be correlated to methylation and another small proportion was correlated to copy number changes seen in CRC. Although methylation status of many loci could not explain the functional relevance to gene expression, these promoters methylation may be used clinically as biomarkers.

## Conclusions

Our genome-wide methylation study in CRC clearly supports most of the previous findings from the literature, and in addition to that we found a large number of novel DML in CRC tissue, some of which may be used for clinical application. Further study is warranted to confirm these findings.

## Authors' contributions

MGK conceived and designed the study, performed data analysis and wrote the manuscript, FJ designed and carried out the genome-wide methylation assay and drafted the manuscript, MR collected the tissue samples and did the histopathology, SR processed the tissue samples and carried out the gene expression and validation assay, RPB processed the tissue samples, and helped in methylation microarray and high density SNP array; RR and CD helped in manuscript, MRZ helped in sample collection and transportation of the samples to USA, MK organized & supervised the tissue collection and was responsible for histopathology, HA helped in manuscript, supported and coordinated the study. All authors read and approved the final manuscript.

## Pre-publication history

The pre-publication history for this paper can be accessed here:

http://www.biomedcentral.com/1755-8794/4/50/prepub

## Supplementary Material

Additional File 1 **Table S1: Patient characteristics**. **Table S2**: Result from Gene Set Enrichment Analysis (GSEA) and GO-ANOVA. **Table S3**: Differentially methylated loci (DML) in CRC compared to adjacent normal colonic mucosa. **Table S4**: Validation of microarray methylation data by qPCR-based methyl profiler assay of twelve genes in paired samples from 10 patients (20 samples). Click here for file

Additional File 2 **Figure S1**: **Electropherogram of DNA samples**. Agilent 2100 BioAnalyzer electropherogram of 10 DNA samples (in different colors) overlaid on ladder marker (shown in violet). Size (bp) of each peak of the DNA ladder in shown on the top of each peak. The figure shows DNA fragment size >10000 bp. Click here for file

Additional File 3 **Figure S2**: **Volcano plot showing methylation status of previously reported genes in our samples**. The Delta β is shown on x-axis and ANOVA p-value on the y-axis. **A**: represents the 245 loci covering the previously reported genes mainly from candidate gene approach-based studies; **B**: represents the 376 loci covering the 132 genes reported from a single study based on genome-wide approach (although testing only 1505 CpG sites). The side bar shows the color scale depending on Delta β where blue indicates hypomethylation and red indicates hypermethylation in CRC. Click here for file

Additional File 4 **Figure S3**: **Heatmap of 14 loci that are differentially methylated in proximal CRC compared to distal CRC**. Unsupervised hierarchical clustering of the 14 loci (rows) in 24 CRC samples (columns). Thirteen of these loci were hypermethylated in proximal CRC compared to distal CRC. The two major clusters generated (top dendogram) in this analysis separated most of the proximal CRC tissues from the distal CRC tissues. Age at diagnosis (>45 yrs or = <45 yrs and the differentiation of the tumor (moderately differentiated or poorly differentiated) are shown above the heatmap. Click here for file

Additional File 5 **Figure S4. Comparison between q-PCR and microarray methylation data**. Graphs are shown for the 12 genes validated by q-PCR. The y-axis plots the β value from microarray data. The x-axis plots the proportion of intermediately methylated and hypermethylated DNA. Click here for file
