# Supplementary material for: A genome-wide DNA methylation study in colorectal carcinoma
Source: BMC Med Genomics. 2011 Jun 23;4:50. doi: 10.1186/1755-8794-4-50 (PMC3135506; doi:10.1186/1755-8794-4-50)
Supplement: Additional File 1 — Table S1: Patient characteristics. Table S2: Result from Gene Set Enrichment Analysis (GSEA) and GO-ANOVA. Table S3: Differentially methylated loci (DML) in CRC compared to adjacent normal colonic mucosa. Table S4: Validation of microarray methylation data by qPCR-based methyl profiler assay of twelve genes in paired samples from 10 patients (20 samples). [file 1755-8794-4-50-S1.DOC]

| **Additional File 1 Table S1**  **Patient characteristics:** | |
| --- | --- |
| Patients: | 24 (m=17, f=7) |
| Age: | 45.54 (SD 16.8) |
| BMI: | 19.56 (SD 3.22) |
| Dx: | Adenocarcinoma: 18 |
|  | Mucinous AdenoCarcinoma: 5 |
| Site: | Distal colon 17, Proximal colon 7 |
| Differentiation: | Moderate 18, Poor 6 |
| TNM classification: |  |
| T2N0Mx: | 7 |
| T2N1Mx: | 1 |
| T3N0Mx: | 6 |
| T3N1Mx: | 5 |
| T3N2Mx: | 4 |
| T4N0Mx: | 1 |

| **Additional File 1 Table S2**  **Result from Gene Set Enrichment Analysis (GSEA) and GO-ANOVA** | | | | | |
| --- | --- | --- | --- | --- | --- |
|
| Gene Set# | Gene Set Description | ES | NES | GO-ANOVA p-value* | DeltaBeta |
| 7193 | inhibition of adenylate cyclase activity by G-protein signaling | 0.66 | 2.04 | 4.03E-08 | 0.10 |
| 21522 | spinal cord motor neuron differentiation | 0.92 | 2.00 | 8.05E-07 | 0.16 |
| 30676 | Rac guanyl-nucleotide exchange factor activity | 0.86 | 1.99 | 5.97E-08 | 0.21 |
| 15277 | kainate selective glutamate receptor activity | 0.84 | 1.88 | 2.84E-07 | 0.14 |
| 48385 | regulation of retinoic acid receptor signaling pathway | 0.73 | 1.86 | 3.94E-10 | 0.18 |
| 5623 | cell | 0.93 | 1.85 | 7.40E-09 | 0.14 |
| 31290 | retinal ganglion cell axon guidance | 0.72 | 1.83 | 1.83E-06 | 0.13 |
| 34185 | apolipoprotein binding | 0.72 | 1.83 | 3.50E-07 | 0.11 |
| 19911 | structural constituent of myelin sheath | 0.90 | 1.83 | 3.03E-07 | 0.19 |
| 4966 | galanin receptor activity | 0.83 | 1.82 | 1.49E-06 | 0.15 |
| 43415 | positive regulation of skeletal muscle regeneration | 0.72 | 1.81 | 7.65E-06 | 0.12 |
| 51890 | regulation of cardioblast differentiation | 0.89 | 1.79 | 3.80E-08 | 0.20 |
| 51891 | positive regulation of cardioblast differentiation | 0.89 | 1.79 | 3.80E-08 | 0.20 |
| 5883 | neurofilament | 0.87 | 1.79 | 4.26E-07 | 0.15 |
| 60080 | regulation of inhibitory postsynaptic membrane potential | 0.79 | 1.78 | 1.79E-06 | 0.13 |
| 60052 | neurofilament cytoskeleton organization | 0.80 | 1.78 | 5.90E-07 | 0.13 |
| 16081 | synaptic vesicle docking during exocytosis | 0.81 | 1.78 | 2.78E-06 | 0.17 |
| 48699 | generation of neurons | 0.74 | 1.78 | 6.16E-05 | 0.10 |
| 48386 | positive regulation of retinoic acid receptor signaling pathway | 0.90 | 1.77 | 7.73E-10 | 0.22 |
| 43392 | negative regulation of DNA binding | 0.72 | 1.77 | 1.29E-08 | 0.12 |
| 9950 | dorsal/ventral axis specification | 0.67 | 1.77 | 2.07E-10 | 0.15 |
| 6776 | vitamin A metabolic process | 0.78 | 1.74 | 3.05E-04 | 0.12 |
| 3828 | alpha-N-acetylneuraminate alpha-2,8-sialyltransferase activity | 0.90 | 1.74 | 4.37E-07 | 0.19 |
| 42573 | retinoic acid metabolic process | 0.67 | 1.74 | 5.82E-07 | 0.15 |
| 55081 | anion homeostasis | 0.68 | 1.74 | 2.36E-07 | 0.13 |
| 55103 | ligase regulator activity | 0.85 | 1.74 | 5.31E-07 | 0.16 |
| 55105 | ubiquitin-protein ligase inhibitor activity | 0.85 | 1.74 | 5.31E-07 | 0.16 |
| 55106 | ubiquitin-protein ligase regulator activity | 0.85 | 1.74 | 5.31E-07 | 0.16 |
| 4983 | neuropeptide Y receptor activity | 0.72 | 1.73 | 9.88E-07 | 0.13 |
| 48676 | axon extension involved in development | 0.82 | 1.73 | 1.28E-07 | 0.17 |
| 35284 | brain segmentation | 0.95 | 1.72 | 1.95E-08 | 0.27 |
| 7379 | segment specification | 0.60 | 1.72 | 5.43E-08 | 0.12 |
| 35020 | regulation of Rac protein signal transduction | 0.87 | 1.71 | 1.25E-07 | 0.16 |
| 45995 | regulation of embryonic development | 0.73 | 1.71 | 4.38E-07 | 0.13 |
| 14056 | regulation of acetylcholine secretion | 0.90 | 1.71 | 3.23E-07 | 0.22 |
| 10389 | regulation of G2/M transition of mitotic cell cycle | 0.87 | 1.69 | 3.83E-07 | 0.17 |
| 7518 | myoblast cell fate determination | 0.88 | 1.68 | 4.96E-06 | 0.17 |
| 21680 | cerebellar Purkinje cell layer development | 0.71 | 1.68 | 2.24E-07 | 0.16 |
| 32281 | alpha-amino-3-hydroxy-5-methyl-4-isoxazolepropionic acid selective glutamate receptor complex | 0.82 | 1.68 | 2.15E-07 | 0.13 |
| 6929 | substrate-bound cell migration | 0.81 | 1.68 | 4.16E-06 | 0.14 |
| 51957 | positive regulation of amino acid transport | 0.89 | 1.67 | 4.21E-06 | 0.11 |
| 14054 | positive regulation of gamma-aminobutyric acid secretion | 0.89 | 1.67 | 4.21E-06 | 0.11 |
| 21985 | neurohypophysis development | 0.92 | 1.67 | 1.86E-04 | 0.11 |
| 4962 | endothelin receptor activity | 0.73 | 1.65 | 4.11E-08 | 0.16 |
| 21530 | spinal cord oligodendrocyte cell fate specification | 0.85 | 1.65 | 1.69E-04 | 0.11 |
| 21778 | oligodendrocyte cell fate specification | 0.85 | 1.65 | 1.69E-04 | 0.11 |
| 21780 | glial cell fate specification | 0.85 | 1.65 | 1.69E-04 | 0.11 |
| 51665 | membrane raft localization | 0.81 | 1.64 | 4.89E-08 | 0.20 |
| 1766 | membrane raft polarization | 0.81 | 1.64 | 4.89E-08 | 0.20 |
| 31580 | membrane raft distribution | 0.81 | 1.64 | 4.89E-08 | 0.20 |
| 32983 | kainate selective glutamate receptor complex | 0.88 | 1.64 | 3.74E-07 | 0.17 |
| 46031 | ADP metabolic process | 0.86 | 1.64 | 5.38E-04 | 0.15 |
| 9179 | purine ribonucleoside diphosphate metabolic process | 0.86 | 1.64 | 5.38E-04 | 0.15 |
| 9185 | ribonucleoside diphosphate metabolic process | 0.86 | 1.64 | 5.38E-04 | 0.15 |
| 32229 | negative regulation of synaptic transmission, GABAergic | 0.84 | 1.62 | 5.94E-07 | 0.14 |
| 4995 | tachykinin receptor activity | 0.74 | 1.61 | 1.72E-06 | 0.18 |
| 32100 | positive regulation of appetite | 0.98 | 1.59 | 6.21E-09 | 0.26 |
| 9190 | cyclic nucleotide biosynthetic process | 0.92 | 1.58 | 6.52E-09 | 0.32 |
| 51153 | regulation of striated muscle cell differentiation | 0.80 | 1.57 | 4.91E-04 | 0.10 |
| 4971 | alpha-amino-3-hydroxy-5-methyl-4-isoxazole propionate selective glutamate receptor activity | 0.95 | 1.54 | 4.51E-08 | 0.18 |
| 30948 | negative regulation of vascular endothelial growth factor receptor signaling pathway | 0.91 | 1.54 | 1.14E-04 | 0.14 |
| 45176 | apical protein localization | 0.70 | 1.54 | 1.13E-06 | 0.11 |
| 42133 | neurotransmitter metabolic process | 0.66 | 1.54 | 3.58E-07 | 0.15 |
| 30284 | estrogen receptor activity | 0.84 | 1.53 | 7.73E-10 | 0.22 |
| 1919 | regulation of receptor recycling | 0.83 | 1.53 | 1.43E-05 | 0.16 |
| 19860 | uracil metabolic process | 0.83 | 1.52 | 1.38E-04 | 0.11 |
| 6210 | thymine catabolic process | 0.83 | 1.52 | 1.38E-04 | 0.11 |
| 6212 | uracil catabolic process | 0.83 | 1.52 | 1.38E-04 | 0.11 |
| 21546 | rhombomere development | 0.63 | 1.52 | 1.58E-05 | 0.14 |
| 50883 | musculoskeletal movement, spinal reflex action | 0.75 | 1.52 | 2.27E-06 | 0.13 |
| 16623 | oxidoreductase activity, acting on the aldehyde or oxo group of donors, oxygen as acceptor | 0.93 | 1.51 | 2.67E-03 | 0.17 |
| 4031 | aldehyde oxidase activity | 0.93 | 1.51 | 2.67E-03 | 0.17 |
| 10466 | negative regulation of peptidase activity | -0.73 | -1.51 | 3.05E-07 | -0.10 |
| 44270 | nitrogen compound catabolic process | -0.79 | -1.51 | 1.02E-05 | -0.13 |
| 30345 | structural constituent of tooth enamel | -0.92 | -1.63 | 7.71E-07 | -0.10 |
| 42953 | lipoprotein transport | -0.75 | -1.66 | 1.04E-07 | -0.11 |
| 15037 | peptide disulfide oxidoreductase activity | -0.80 | -1.67 | 1.10E-06 | -0.13 |
| 15038 | glutathione disulfide oxidoreductase activity | -0.80 | -1.67 | 1.10E-06 | -0.13 |
| 30492 | hemoglobin binding | -0.81 | -1.69 | 7.54E-08 | -0.11 |
| 43031 | negative regulation of macrophage activation | -0.90 | -1.73 | 7.85E-08 | -0.15 |
| 55078 | sodium ion homeostasis | -0.77 | -1.79 | 9.76E-07 | -0.11 |
| 19864 | IgG binding | -0.73 | -1.79 | 7.35E-08 | -0.15 |
| 30851 | granulocyte differentiation | -0.85 | -1.80 | 8.31E-09 | -0.11 |
| 51183 | vitamin transporter activity | -0.73 | -1.83 | 1.01E-08 | -0.11 |
| 6555 | methionine metabolic process | -0.85 | -1.83 | 2.22E-07 | -0.14 |
| 5132 | interferon-alpha/beta receptor binding | -0.82 | -1.84 | 2.39E-05 | -0.12 |
| 17040 | ceramidase activity | -0.72 | -1.98 | 1.14E-06 | -0.10 |
|  |  |  |  |  |  |
| The "gene sets" are sorted by NES in descending order. These "gene sets" had p=<0.01 for ES, NES either =<-1.5 or >=1.5 in GSEA and also showed significant GO-ANOVA p-value at FRD 0.01 and an average delta beta =<-0.1 or >=0.1. ES: Enrichment Score; NES: Normalized Enrichment Score; positive NES indicates hypermethylation and negative NES indicates hypomethylation;  * GO-ANOVA p-value adjusted for sex, person-to-parson variation and location of tumor. # for gene list within gene set: http://www.geneontology.org | | | | | |

| **Additional File 1 Table S3**  **Differentially methylated loci (DML) in CRC compared to adjacent normal colonic mucosa.** | | | | | | | | | | |
| --- | --- | --- | --- | --- | --- | --- | --- | --- | --- | --- |
| Probeset ID | GENE_ID | SYMBOL | CHR | DISTANCE  TO_TSS | p-value (Tissue)* | Fold Change | Diff. Methylation | Delta Beta | PC_tissue** | Type*** |
| cg22029275 | GeneID:219287 | *FLJ25477* | 13 | 73 | 6.25E-11 | 4.48 | CRC Hypermethylated | 0.543 | 69.131 | Novel |
| cg20415809 | GeneID:3676 | *ITGA4* | 2 | 236 | 4.51E-12 | 5.37 | CRC Hypermethylated | 0.542 | 73.012 | Previously reported |
| cg14063008 | GeneID:153090 | *DAB2IP* | 9 | 50 | 1.81E-07 | 14.47 | CRC Hypermethylated | 0.530 | 51.594 | Previously reported |
| cg27650175 | GeneID:153090 | *DAB2IP* | 9 | 9 | 4.46E-08 | 107.86 | CRC Hypermethylated | 0.523 | 55.575 | Previously reported |
| cg24687051 | GeneID:56479 | *KCNQ5* | 6 | 238 | 2.98E-08 | 3.35 | CRC Hypermethylated | 0.522 | 60.064 | Novel |
| cg17892556 | GeneID:90589 | *ZNF625* | 19 | 65 | 1.79E-07 | 5.87 | CRC Hypermethylated | 0.513 | 51.625 | Novel |
| cg24794433 | GeneID:153090 | *DAB2IP* | 9 | 523 | 3.23E-08 | 7.01 | CRC Hypermethylated | 0.507 | 58.013 | Previously reported |
| cg06744574 | GeneID:79656 | *C1orf165* | 1 | 144 | 5.45E-09 | 4.31 | CRC Hypermethylated | 0.505 | 56.407 | Novel |
| cg13577076 | GeneID:5575 | *PRKAR1B* | 7 | 323 | 3.25E-11 | 2.78 | CRC Hypermethylated | 0.485 | 68.602 | Novel |
| cg05345286 | GeneID:4188 | *MDFI* | 6 | 388 | 5.72E-11 | 5.31 | CRC Hypermethylated | 0.484 | 71.437 | Novel |
| cg07080358 | GeneID:25927 | *C2orf32* | 2 | 336 | 4.60E-11 | 3.04 | CRC Hypermethylated | 0.478 | 67.773 | Novel |
| cg11657808 | GeneID:6262 | *RYR2* | 1 | 248 | 2.69E-09 | 3.46 | CRC Hypermethylated | 0.456 | 64.307 | Novel |
| cg17872757 | GeneID:2313 | *FLI1* | 11 | 191 | 9.93E-08 | 52.78 | CRC Hypermethylated | 0.452 | 53.286 | Previously reported |
| cg05684891 | GeneID:153090 | *DAB2IP* | 9 | 0 | 9.50E-07 | 21.53 | CRC Hypermethylated | 0.445 | 49.057 | Previously reported |
| cg08128768 | GeneID:153090 | *DAB2IP* | 9 | 533 | 1.77E-08 | 5.60 | CRC Hypermethylated | 0.442 | 58.165 | Previously reported |
| cg08383315 | GeneID:79608 | *RIC3* | 11 | 7 | 2.63E-10 | 2.82 | CRC Hypermethylated | 0.441 | 66.850 | Novel |
| cg03853987 | GeneID:9486 | *CHST10* | 2 | 188 | 7.09E-07 | 5.98 | CRC Hypermethylated | 0.436 | 48.468 | Novel |
| cg26309134 | GeneID:147947 | *ZNF542* | 19 | 63 | 2.31E-08 | 2.99 | CRC Hypermethylated | 0.422 | 50.885 | Novel |
| cg07981910 | GeneID:153090 | *DAB2IP* | 9 | 0 | 1.36E-07 | 26.15 | CRC Hypermethylated | 0.422 | 53.802 | Previously reported |
| cg08190044 | GeneID:57198 | *ATP8B2* | 1 | 299 | 1.17E-07 | 9.22 | CRC Hypermethylated | 0.419 | 55.224 | Novel |
| cg01683883 | GeneID:146225 | *CMTM2* | 16 | 298 | 9.71E-09 | 2.51 | CRC Hypermethylated | 0.402 | 57.574 | Novel |
| cg19118812 | GeneID:9844 | *ELMO1* | 7 | 18 | 2.77E-06 | 6.60 | CRC Hypermethylated | 0.399 | 42.537 | Novel |
| cg16787600 | GeneID:22986 | *SORCS3* | 10 | 21 | 2.14E-08 | 2.34 | CRC Hypermethylated | 0.399 | 59.436 | Novel |
| cg13060997 | GeneID:8900 | *CCNA1* | 13 | 22 | 1.47E-08 | 2.89 | CRC Hypermethylated | 0.398 | 52.804 | Previously reported |
| cg04034767 | GeneID:160622 | *GRASP* | 12 | 159 | 9.35E-06 | 5.50 | CRC Hypermethylated | 0.398 | 40.704 | Novel |
| cg24446548 | GeneID:7291 | *TWIST1* | 7 | 32 | 6.46E-06 | 4.78 | CRC Hypermethylated | 0.394 | 39.070 | Previously reported |
| cg13801416 | GeneID:231 | *AKR1B1* | 7 | 31 | 4.79E-06 | 5.58 | CRC Hypermethylated | 0.390 | 39.316 | Novel |
| cg15731815 | GeneID:148646 | *C1orf188* | 1 | 340 | 4.26E-08 | 2.54 | CRC Hypermethylated | 0.389 | 57.115 | Novel |
| cg13060154 | GeneID:153090 | *DAB2IP* | 9 | 0 | 5.94E-08 | 3.40 | CRC Hypermethylated | 0.383 | 55.783 | Previously reported |
| cg00929855 | GeneID:3303 | *HSPA1A* | 6 | 27 | 2.77E-06 | 4.44 | CRC Hypermethylated | 0.382 | 43.562 | Novel |
| cg23297477 | GeneID:123920 | *CMTM3* | 16 | 80 | 5.18E-08 | 2.78 | CRC Hypermethylated | 0.382 | 46.460 | Novel |
| cg18755783 | GeneID:23111 | *SPG20* | 13 | 548 | 1.63E-07 | 2.91 | CRC Hypermethylated | 0.380 | 50.698 | Novel |
| cg20286200 | GeneID:2070 | *EYA4* | 6 | 246 | 2.71E-08 | 3.14 | CRC Hypermethylated | 0.380 | 52.519 | Previously reported |
| cg06995715 | GeneID:2300 | *FOXL1* | 16 | 260 | 4.41E-09 | 2.20 | CRC Hypermethylated | 0.380 | 55.601 | Novel |
| cg05624932 | GeneID:83690 | *CRISPLD1* | 8 | 334 | 7.09E-08 | 3.39 | CRC Hypermethylated | 0.380 | 51.196 | Novel |
| cg07104706 | GeneID:114798 | *SLITRK1* | 13 | 401 | 1.28E-08 | 2.29 | CRC Hypermethylated | 0.379 | 52.530 | Novel |
| cg13562911 | GeneID:54898 | *ELOVL2* | 6 | 471 | 3.06E-06 | 4.13 | CRC Hypermethylated | 0.374 | 39.775 | Novel |
| cg24562819 | GeneID:7056 | *THBD* | 20 | 495 | 6.16E-08 | 2.88 | CRC Hypermethylated | 0.374 | 41.550 | Novel |
| cg17190608 | GeneID:23316 | *CUTL2* | 12 | 340 | 3.07E-07 | 3.87 | CRC Hypermethylated | 0.373 | 48.626 | Novel |
| cg19456540 | GeneID:4990 | *SIX6* | 14 | 263 | 6.88E-09 | 2.67 | CRC Hypermethylated | 0.370 | 60.493 | Novel |
| cg12571423 | GeneID:8900 | *CCNA1* | 13 | 115 | 1.10E-08 | 2.14 | CRC Hypermethylated | 0.369 | 51.233 | Previously reported |
| cg21359747 | GeneID:220 | *ALDH1A3* | 15 | 575 | 2.05E-07 | 3.14 | CRC Hypermethylated | 0.369 | 46.735 | Novel |
| cg01805282 | GeneID:2070 | *EYA4* | 6 | 0 | 3.31E-09 | 2.30 | CRC Hypermethylated | 0.366 | 59.947 | Previously reported |
| cg01009664 | GeneID:7200 | *TRH* | 3 | 50 | 4.77E-11 | 2.17 | CRC Hypermethylated | 0.364 | 66.840 | Novel |
| cg15202954 | GeneID:259232 | *VGCNL1* | 13 | 290 | 1.12E-10 | 1.97 | CRC Hypermethylated | 0.364 | 71.792 | Novel |
| cg16335762 | GeneID:123920 | *CMTM3* | 16 | 294 | 7.03E-08 | 2.18 | CRC Hypermethylated | 0.363 | 43.341 | Novel |
| cg14896516 | GeneID:1395 | *CRHR2* | 7 | 465 | 3.34E-07 | 4.21 | CRC Hypermethylated | 0.363 | 48.296 | Novel |
| cg24662718 | GeneID:10451 | *VAV3* | 1 | 70 | 5.09E-06 | 3.73 | CRC Hypermethylated | 0.362 | 39.561 | Novel |
| cg01555431 | GeneID:9590 | *AKAP12* | 6 | 517 | 6.36E-09 | 2.50 | CRC Hypermethylated | 0.362 | 54.066 | Novel |
| cg27652350 | GeneID:220 | *ALDH1A3* | 15 | 0 | 3.68E-09 | 2.37 | CRC Hypermethylated | 0.361 | 50.971 | Novel |
| cg12508624 | GeneID:7070 | *THY1* | 11 | 100 | 5.78E-08 | 3.36 | CRC Hypermethylated | 0.361 | 52.961 | Previously reported |
| cg03168582 | GeneID:1761 | *DMRT1* | 9 | 160 | 3.13E-06 | 3.49 | CRC Hypermethylated | 0.356 | 38.446 | Novel |
| cg20330472 | GeneID:2070 | *EYA4* | 6 | 0 | 2.91E-06 | 4.02 | CRC Hypermethylated | 0.356 | 43.390 | Previously reported |
| cg18671950 | GeneID:2200 | *FBN1* | 15 | 146 | 3.53E-07 | 3.00 | CRC Hypermethylated | 0.352 | 53.144 | Novel |
| cg26656135 | GeneID:2070 | *EYA4* | 6 | 261 | 7.17E-11 | 3.11 | CRC Hypermethylated | 0.351 | 70.118 | Previously reported |
| cg20253551 | GeneID:2099 | *ESR1* | 6 | 0 | 1.04E-08 | 4.76 | CRC Hypermethylated | 0.350 | 55.528 | Previously reported |
| cg23054883 | GeneID:11211 | *FZD10* | 12 | 548 | 4.56E-09 | 2.04 | CRC Hypermethylated | 0.350 | 58.380 | Novel |
| cg12880658 | GeneID:1036 | *CDO1* | 5 | 19 | 1.22E-08 | 2.87 | CRC Hypermethylated | 0.349 | 55.457 | Novel |
| cg22879515 | GeneID:54766 | *BTG4* | 11 | 451 | 1.01E-05 | 3.20 | CRC Hypermethylated | 0.349 | 39.171 | Novel |
| cg17525406 | GeneID:55966 | *AJAP1* | 1 | 415 | 7.86E-07 | 1.99 | CRC Hypermethylated | 0.348 | 44.985 | Novel |
| cg19784477 | GeneID:7980 | *TFPI2* | 7 | 0 | 1.31E-07 | 4.05 | CRC Hypermethylated | 0.347 | 49.672 | Previously reported |
| cg04784672 | GeneID:145581 | *LRFN5* | 14 | 334 | 6.41E-08 | 2.62 | CRC Hypermethylated | 0.347 | 53.201 | Novel |
| cg10293925 | GeneID:273 | *AMPH* | 7 | 35 | 3.15E-06 | 2.16 | CRC Hypermethylated | 0.347 | 48.982 | Novel |
| cg12741420 | GeneID:3662 | *IRF4* | 6 | 371 | 3.06E-09 | 7.54 | CRC Hypermethylated | 0.346 | 57.831 | Novel |
| cg21226224 | GeneID:64321 | *SOX17* | 8 | 324 | 2.51E-07 | 2.66 | CRC Hypermethylated | 0.345 | 48.910 | Previously reported |
| cg13823136 | GeneID:81849 | *ST6GALNAC5* | 1 | 48 | 2.01E-08 | 4.03 | CRC Hypermethylated | 0.343 | 54.685 | Novel |
| cg27188703 | GeneID:23017 | *FAIM2* | 12 | 139 | 6.68E-06 | 2.19 | CRC Hypermethylated | 0.342 | 42.260 | Novel |
| cg03538436 | GeneID:4842 | *NOS1* | 12 | 212 | 5.31E-07 | 2.24 | CRC Hypermethylated | 0.342 | 46.352 | Novel |
| cg20959866 | GeneID:55966 | *AJAP1* | 1 | 1097 | 2.79E-07 | 2.03 | CRC Hypermethylated | 0.341 | 56.359 | Novel |
| cg08090772 | GeneID:137872 | *ADHFE1* | 8 | 94 | 3.98E-07 | 3.49 | CRC Hypermethylated | 0.340 | 50.840 | Novel |
| cg18239753 | GeneID:202559 | *KHDRBS2* | 6 | 169 | 3.38E-07 | 2.05 | CRC Hypermethylated | 0.339 | 51.581 | Novel |
| cg24176563 | GeneID:2070 | *EYA4* | 6 | 263 | 3.79E-10 | 3.19 | CRC Hypermethylated | 0.338 | 68.331 | Previously reported |
| cg25764191 | GeneID:9118 | *INA* | 10 | 295 | 3.76E-06 | 2.41 | CRC Hypermethylated | 0.338 | 41.579 | Novel |
| cg11939071 | GeneID:1840 | *DTX1* | 12 | 1233 | 2.57E-08 | 2.87 | CRC Hypermethylated | 0.338 | 55.712 | Novel |
| cg07846167 | GeneID:54751 | *FBLIM1* | 1 | 497 | 1.26E-06 | 2.60 | CRC Hypermethylated | 0.337 | 53.955 | Novel |
| cg22598028 | GeneID:285349 | *ZNF660* | 3 | 36 | 3.03E-06 | 2.58 | CRC Hypermethylated | 0.337 | 40.210 | Novel |
| cg05158615 | GeneID:4852 | *NPY* | 7 | 250 | 2.46E-09 | 2.33 | CRC Hypermethylated | 0.335 | 55.374 | Previously reported |
| cg16761581 | GeneID:196883 | *ADCY4* | 14 | 57 | 3.08E-06 | 3.42 | CRC Hypermethylated | 0.334 | 37.596 | Novel |
| cg18416881 | GeneID:231 | *AKR1B1* | 7 | 65 | 9.16E-06 | 5.16 | CRC Hypermethylated | 0.334 | 37.957 | Novel |
| cg15540820 | GeneID:8320 | *EOMES* | 3 | 1498 | 7.66E-09 | 2.18 | CRC Hypermethylated | 0.334 | 57.104 | Novel |
| cg20881910 | GeneID:50863 | *HNT* | 11 | 380 | 9.06E-08 | 1.99 | CRC Hypermethylated | 0.333 | 58.528 | Novel |
| cg11500797 | GeneID:1749 | *DLX5* | 7 | 0 | 8.67E-08 | 2.32 | CRC Hypermethylated | 0.333 | 50.087 | Novel |
| cg14785479 | GeneID:91179 | *SCARF2* | 22 | 389 | 2.13E-06 | 1.94 | CRC Hypermethylated | 0.332 | 44.104 | Novel |
| cg26162582 | GeneID:3742 | *KCNA6* | 12 | 49 | 2.44E-06 | 2.92 | CRC Hypermethylated | 0.332 | 42.355 | Novel |
| cg26590537 | GeneID:3736 | *KCNA1* | 12 | 343 | 2.18E-07 | 3.86 | CRC Hypermethylated | 0.331 | 48.453 | Novel |
| cg19292008 | GeneID:2304 | *FOXE1* | 9 | 259 | 1.01E-06 | 3.56 | CRC Hypermethylated | 0.330 | 48.089 | Novel |
| cg13877915 | GeneID:7691 | *ZNF132* | 19 | 83 | 1.54E-06 | 3.64 | CRC Hypermethylated | 0.329 | 38.688 | Novel |
| cg25216696 | GeneID:2626 | *GATA4* | 8 | 0 | 9.37E-10 | 2.21 | CRC Hypermethylated | 0.329 | 54.274 | Previously reported |
| cg21210758 | GeneID:2121 | *EVC* | 4 | 81 | 2.27E-08 | 2.15 | CRC Hypermethylated | 0.329 | 50.168 | Novel |
| cg20209009 | GeneID:30009 | *TBX21* | 17 | 712 | 3.93E-07 | 3.92 | CRC Hypermethylated | 0.327 | 50.315 | Novel |
| cg22799321 | GeneID:7980 | *TFPI2* | 7 | 0 | 9.75E-08 | 3.95 | CRC Hypermethylated | 0.326 | 51.857 | Previously reported |
| cg03388193 | GeneID:60495 | *HPSE2* | 10 | 451 | 8.71E-08 | 3.23 | CRC Hypermethylated | 0.326 | 47.927 | Novel |
| cg12265829 | GeneID:196883 | *ADCY4* | 14 | 158 | 1.62E-06 | 2.84 | CRC Hypermethylated | 0.326 | 41.930 | Novel |
| cg23141855 | GeneID:7980 | *TFPI2* | 7 | 173 | 6.20E-09 | 3.85 | CRC Hypermethylated | 0.326 | 57.894 | Previously reported |
| cg17296166 | GeneID:55228 | *FLJ10781* | 19 | 210 | 1.88E-05 | 3.62 | CRC Hypermethylated | 0.326 | 38.506 | Novel |
| cg18236079 | GeneID:9955 | *HS3ST3A1* | 17 | 346 | 1.03E-06 | 3.48 | CRC Hypermethylated | 0.326 | 46.772 | Novel |
| cg12582959 | GeneID:29903 | *HSU79303* | 19 | 202 | 7.68E-09 | 1.86 | CRC Hypermethylated | 0.325 | 62.283 | Novel |
| cg08453021 | GeneID:9844 | *ELMO1* | 7 | 119 | 3.32E-07 | 4.50 | CRC Hypermethylated | 0.325 | 45.537 | Novel |
| cg07903918 | GeneID:9568 | *GABBR2* | 9 | 507 | 4.41E-07 | 2.17 | CRC Hypermethylated | 0.324 | 46.585 | Novel |
| cg26831415 | GeneID:286097 | *EFHA2* | 8 | 522 | 8.35E-07 | 3.02 | CRC Hypermethylated | 0.324 | 45.282 | Novel |
| cg03064067 | GeneID:55117 | *SLC6A15* | 12 | 342 | 2.44E-08 | 2.08 | CRC Hypermethylated | 0.323 | 54.043 | Novel |
| cg17371081 | GeneID:4745 | *NELL1* | 11 | 179 | 6.91E-08 | 2.24 | CRC Hypermethylated | 0.322 | 51.059 | Novel |
| cg23686014 | GeneID:7980 | *TFPI2* | 7 | 141 | 2.28E-08 | 3.50 | CRC Hypermethylated | 0.322 | 53.485 | Previously reported |
| cg17108819 | GeneID:925 | *CD8A* | 2 | 114 | 1.87E-05 | 4.40 | CRC Hypermethylated | 0.319 | 35.865 | Novel |
| cg10918202 | GeneID:79870 | *BAALC* | 8 | 252 | 2.73E-05 | 3.59 | CRC Hypermethylated | 0.319 | 33.476 | Novel |
| cg09871315 | GeneID:3199 | *HOXA2* | 7 | 288 | 1.86E-07 | 1.71 | CRC Hypermethylated | 0.319 | 54.144 | Novel |
| cg25574024 | GeneID:51214 | *IGF2AS* | 11 | 147 | 1.18E-06 | 2.06 | CRC Hypermethylated | 0.318 | 45.761 | Previously reported |
| cg25307902 | GeneID:6616 | *SNAP25* | 20 | 267 | 7.47E-06 | 9.05 | CRC Hypermethylated | 0.317 | 40.301 | Novel |
| cg18277754 | GeneID:238 | *ALK* | 2 | 280 | 1.79E-07 | 2.73 | CRC Hypermethylated | 0.316 | 50.153 | Previously reported |
| cg15156078 | GeneID:5923 | *RASGRF1* | 15 | 573 | 9.88E-08 | 3.19 | CRC Hypermethylated | 0.316 | 52.784 | Previously reported |
| cg19246110 | GeneID:79891 | *ZNF671* | 19 | 55 | 2.96E-06 | 3.02 | CRC Hypermethylated | 0.315 | 30.622 | Novel |
| cg26069745 | GeneID:3199 | *HOXA2* | 7 | 194 | 7.96E-09 | 1.66 | CRC Hypermethylated | 0.313 | 61.026 | Novel |
| cg12832649 | GeneID:6695 | *SPOCK* | 5 | 132 | 1.63E-05 | 2.52 | CRC Hypermethylated | 0.313 | 38.132 | Novel |
| cg15802898 | GeneID:2304 | *FOXE1* | 9 | 336 | 4.33E-08 | 3.18 | CRC Hypermethylated | 0.312 | 52.609 | Novel |
| cg15087147 | GeneID:64101 | *LRRC4* | 7 | 1167 | 3.34E-07 | 7.81 | CRC Hypermethylated | 0.312 | 52.306 | Novel |
| cg07758904 | GeneID:57864 | *TSCOT* | 9 | 221 | 3.54E-06 | 2.15 | CRC Hypermethylated | 0.311 | 42.851 | Novel |
| cg19774122 | GeneID:3908 | *LAMA2* | 6 | 280 | 3.03E-10 | 1.97 | CRC Hypermethylated | 0.311 | 49.339 | Novel |
| cg23887396 | GeneID:23098 | *SARM1* | 17 | 425 | 6.94E-08 | 4.08 | CRC Hypermethylated | 0.308 | 54.077 | Novel |
| cg18952647 | GeneID:646 | *BNC1* | 15 | 276 | 1.43E-06 | 2.16 | CRC Hypermethylated | 0.308 | 44.911 | Previously reported |
| cg18349835 | GeneID:7434 | *VIPR2* | 7 | 542 | 3.96E-07 | 3.08 | CRC Hypermethylated | 0.307 | 45.826 | Novel |
| cg25465406 | GeneID:3000 | *GUCY2D* | 17 | 110 | 9.74E-06 | 3.20 | CRC Hypermethylated | 0.306 | 42.916 | Novel |
| cg03382304 | GeneID:58494 | *JAM2* | 21 | 587 | 7.82E-07 | 4.10 | CRC Hypermethylated | 0.306 | 47.719 | Novel |
| cg08047907 | GeneID:57821 | *C1orf114* | 1 | 188 | 3.45E-08 | 2.88 | CRC Hypermethylated | 0.306 | 51.086 | Novel |
| cg23694248 | GeneID:5801 | *PTPRR* | 12 | 271 | 7.57E-06 | 2.13 | CRC Hypermethylated | 0.304 | 44.602 | Novel |
| cg20530314 | GeneID:185 | *AGTR1* | 3 | 154 | 0.000131 | 3.46 | CRC Hypermethylated | 0.304 | 30.644 | Previously reported |
| cg09229912 | GeneID:23316 | *CUTL2* | 12 | 134 | 1.33E-07 | 1.72 | CRC Hypermethylated | 0.303 | 45.193 | Novel |
| cg20001829 | GeneID:84866 | *TMEM25* | 11 | 418 | 6.72E-06 | 3.73 | CRC Hypermethylated | 0.303 | 40.100 | Novel |
| cg10520887 | GeneID:8633 | *UNC5C* | 4 | 68 | 1.18E-06 | 2.62 | CRC Hypermethylated | 0.303 | 43.690 | Previously reported |
| cg04391111 | GeneID:7161 | *TP73* | 1 | 0 | 4.83E-05 | 2.76 | CRC Hypermethylated | 0.303 | 39.479 | Novel |
| cg21432954 | GeneID:7223 | *TRPC4* | 13 | 126 | 2.31E-06 | 2.79 | CRC Hypermethylated | 0.303 | 40.467 | Novel |
| cg22471346 | GeneID:8522 | *GAS7* | 17 | 395 | 6.99E-09 | 2.11 | CRC Hypermethylated | 0.302 | 61.017 | Previously reported |
| cg10784030 | GeneID:3633 | *INPP5B* | 1 | 5 | 0.000123 | 3.89 | CRC Hypermethylated | 0.302 | 31.568 | Novel |
| cg01295203 | GeneID:63978 | *PRDM14* | 8 | 637 | 9.57E-06 | 3.18 | CRC Hypermethylated | 0.302 | 37.794 | Novel |
| cg20640433 | GeneID:3908 | *LAMA2* | 6 | 136 | 1.64E-07 | 1.96 | CRC Hypermethylated | 0.302 | 50.112 | Novel |
| cg14893163 | GeneID:27253 | *PCDH17* | 13 | 92 | 5.69E-10 | 2.19 | CRC Hypermethylated | 0.302 | 64.660 | Novel |
| cg00848728 | GeneID:1600 | *DAB1* | 1 | 193 | 7.16E-08 | 3.75 | CRC Hypermethylated | 0.300 | 48.401 | Novel |
| cg16422907 | GeneID:8900 | *CCNA1* | 13 | 101 | 3.48E-09 | 1.93 | CRC Hypermethylated | 0.300 | 57.688 | Previously reported |
| cg21096399 | GeneID:4162 | *MCAM* | 11 | 305 | 2.00E-08 | 1.73 | CRC Hypermethylated | 0.300 | 60.301 | Novel |
| cg25228126 | GeneID:2535 | *FZD2* | 17 | 385 | 1.21E-05 | 2.69 | CRC Hypermethylated | 0.300 | 39.139 | Novel |
| cg01777397 | GeneID:79745 | *RSNL2* | 2 | 372 | 6.69E-08 | 2.58 | CRC Hypermethylated | 0.300 | 44.716 | Novel |
| cg26924825 | GeneID:3931 | *LCAT* | 16 | 150 | 5.40E-07 | 1.67 | CRC Hypermethylated | 0.299 | 47.850 | Novel |
| cg24068372 | GeneID:349136 | *LOC349136* | 7 | 479 | 1.96E-05 | 2.11 | CRC Hypermethylated | 0.299 | 38.078 | Novel |
| cg19343464 | GeneID:2893 | *GRIA4* | 11 | 216 | 3.39E-07 | 2.22 | CRC Hypermethylated | 0.299 | 46.634 | Novel |
| cg13958426 | GeneID:57821 | *C1orf114* | 1 | 33 | 3.15E-06 | 3.35 | CRC Hypermethylated | 0.298 | 42.606 | Novel |
| cg22609784 | GeneID:4487 | *MSX1* | 4 | 0 | 2.30E-10 | 1.92 | CRC Hypermethylated | 0.296 | 55.695 | Previously reported |
| cg20073553 | GeneID:579 | *BAPX1* | 4 | 747 | 1.70E-06 | 2.22 | CRC Hypermethylated | 0.295 | 46.660 | Novel |
| cg19718882 | GeneID:51352 | *WIT-1* | 11 | 761 | 9.33E-09 | 2.19 | CRC Hypermethylated | 0.295 | 49.243 | Novel |
| cg14384532 | GeneID:4916 | *NTRK3* | 15 | 963 | 4.47E-10 | 2.20 | CRC Hypermethylated | 0.295 | 58.013 | Previously reported |
| cg15250797 | GeneID:6344 | *SCTR* | 2 | 382 | 1.91E-07 | 1.86 | CRC Hypermethylated | 0.295 | 55.013 | Novel |
| cg13912117 | GeneID:114 | *ADCY8* | 8 | 117 | 3.41E-08 | 1.58 | CRC Hypermethylated | 0.294 | 53.485 | Novel |
| cg00903242 | GeneID:10752 | *CHL1* | 3 | 489 | 8.15E-07 | 2.29 | CRC Hypermethylated | 0.294 | 46.079 | Novel |
| cg23242898 | GeneID:1630 | *DCC* | 18 | 115 | 2.85E-06 | 1.88 | CRC Hypermethylated | 0.294 | 35.261 | Previously reported |
| cg01580681 | GeneID:9464 | *HAND2* | 4 | 578 | 1.27E-07 | 1.95 | CRC Hypermethylated | 0.294 | 47.488 | Novel |
| cg00116234 | GeneID:92949 | *ADAMTSL1* | 9 | 76 | 1.59E-05 | 4.87 | CRC Hypermethylated | 0.294 | 38.992 | Novel |
| cg13806135 | GeneID:27253 | *PCDH17* | 13 | 899 | 4.23E-06 | 2.58 | CRC Hypermethylated | 0.293 | 35.406 | Novel |
| cg25438963 | GeneID:8352 | *HIST1H3C* | 6 | 24 | 4.50E-05 | 2.62 | CRC Hypermethylated | 0.293 | 30.469 | Novel |
| cg05774801 | GeneID:6423 | *SFRP2* | 4 | 0 | 8.14E-07 | 3.65 | CRC Hypermethylated | 0.293 | 46.112 | Novel |
| cg23898073 | GeneID:2674 | *GFRA1* | 10 | 28 | 2.76E-06 | 2.88 | CRC Hypermethylated | 0.293 | 41.164 | Novel |
| cg27546237 | GeneID:1282 | *COL4A1* | 13 | 994 | 2.98E-07 | 2.57 | CRC Hypermethylated | 0.293 | 53.772 | Novel |
| cg04456238 | GeneID:7490 | *WT1* | 11 | 0 | 1.84E-10 | 1.80 | CRC Hypermethylated | 0.292 | 64.756 | Previously reported |
| cg04534765 | GeneID:2587 | *GALR1* | 18 | 636 | 1.15E-09 | 1.81 | CRC Hypermethylated | 0.292 | 61.949 | Previously reported |
| cg22821324 | GeneID:2823 | *GPM6A* | 4 | 209 | 1.03E-06 | 5.59 | CRC Hypermethylated | 0.292 | 45.019 | Novel |
| cg05500015 | GeneID:10003 | *NAALAD2* | 11 | 63 | 2.03E-07 | 2.32 | CRC Hypermethylated | 0.292 | 44.810 | Novel |
| cg08190291 | GeneID:11096 | *ADAMTS5* | 21 | 610 | 3.49E-06 | 2.47 | CRC Hypermethylated | 0.291 | 41.681 | Novel |
| cg14377593 | GeneID:7980 | *TFPI2* | 7 | 118 | 1.68E-07 | 4.77 | CRC Hypermethylated | 0.291 | 49.329 | Previously reported |
| cg11812218 | GeneID:2693 | *GHSR* | 3 | 717 | 5.70E-07 | 2.71 | CRC Hypermethylated | 0.291 | 46.725 | Novel |
| cg11354906 | GeneID:6423 | *SFRP2* | 4 | 0 | 1.30E-06 | 2.53 | CRC Hypermethylated | 0.290 | 43.305 | Novel |
| cg21243096 | GeneID:5453 | *POU3F1* | 1 | 893 | 1.47E-06 | 2.26 | CRC Hypermethylated | 0.290 | 45.927 | Novel |
| cg15361590 | GeneID:56098 | *PCDHGC4* | 5 | 40 | 1.25E-08 | 2.06 | CRC Hypermethylated | 0.290 | 50.027 | Novel |
| cg18338311 | GeneID:124842 | *LOC124842* | 17 | 171 | 4.02E-05 | 2.45 | CRC Hypermethylated | 0.290 | 30.703 | Novel |
| cg25691167 | GeneID:222894 | *FERD3L* | 7 | 83 | 5.23E-07 | 1.98 | CRC Hypermethylated | 0.289 | 42.661 | Novel |
| cg09083627 | GeneID:26050 | *SLITRK5* | 13 | 273 | 0.000307 | 1.97 | CRC Hypermethylated | 0.289 | 31.962 | Novel |
| cg00891278 | GeneID:348807 | *CCDC37* | 3 | 2 | 8.70E-06 | 1.89 | CRC Hypermethylated | 0.289 | 39.363 | Novel |
| cg23207990 | GeneID:6423 | *SFRP2* | 4 | 0 | 1.08E-05 | 2.04 | CRC Hypermethylated | 0.288 | 42.686 | Novel |
| cg21696393 | GeneID:30812 | *SOX8* | 16 | 1407 | 1.06E-07 | 2.36 | CRC Hypermethylated | 0.288 | 56.514 | Novel |
| cg16340268 | GeneID:3707 | *ITPKB* | 1 | 23 | 1.77E-06 | 5.13 | CRC Hypermethylated | 0.287 | 46.955 | Novel |
| cg13206017 | GeneID:6750 | *SST* | 3 | 24 | 6.05E-06 | 3.24 | CRC Hypermethylated | 0.286 | 35.073 | Novel |
| cg16254309 | GeneID:26047 | *CNTNAP2* | 7 | 323 | 3.56E-08 | 1.69 | CRC Hypermethylated | 0.285 | 51.501 | Novel |
| cg06825142 | GeneID:1815 | *DRD4* | 11 | 135 | 3.40E-05 | 3.27 | CRC Hypermethylated | 0.285 | 34.539 | Novel |
| cg19884262 | GeneID:399823 | *FLJ46831* | 10 | 16 | 1.22E-07 | 1.83 | CRC Hypermethylated | 0.285 | 48.836 | Novel |
| cg02126753 | GeneID:165 | *AEBP1* | 7 | 38 | 0.000209 | 3.01 | CRC Hypermethylated | 0.284 | 29.902 | Novel |
| cg18335068 | GeneID:342926 | *ZNF677* | 19 | 201 | 2.61E-07 | 2.12 | CRC Hypermethylated | 0.284 | 46.685 | Novel |
| cg26128092 | GeneID:49856 | *WDR8* | 1 | 913 | 2.52E-07 | 1.81 | CRC Hypermethylated | 0.284 | 45.148 | Novel |
| cg09551147 | GeneID:22986 | *SORCS3* | 10 | 902 | 0.000233 | 2.62 | CRC Hypermethylated | 0.284 | 29.483 | Novel |
| cg06908778 | GeneID:9576 | *SPAG6* | 10 | 203 | 8.14E-07 | 2.78 | CRC Hypermethylated | 0.284 | 46.475 | Novel |
| cg03038003 | GeneID:79656 | *C1orf165* | 1 | 397 | 0.000156 | 3.75 | CRC Hypermethylated | 0.283 | 28.145 | Novel |
| cg11668844 | GeneID:23263 | *MCF2L* | 13 | 446 | 5.08E-05 | 2.53 | CRC Hypermethylated | 0.283 | 36.570 | Novel |
| cg18765542 | GeneID:8525 | *DGKZ* | 11 | 638 | 5.52E-05 | 2.67 | CRC Hypermethylated | 0.283 | 35.329 | Novel |
| cg19751300 | GeneID:29906 | *ST8SIA5* | 18 | 640 | 3.10E-08 | 3.09 | CRC Hypermethylated | 0.282 | 57.395 | Novel |
| cg18089852 | GeneID:8622 | *PDE8B* | 5 | 344 | 2.04E-06 | 2.41 | CRC Hypermethylated | 0.282 | 45.054 | Novel |
| cg04599297 | GeneID:2572 | *GAD2* | 10 | 152 | 2.09E-07 | 2.63 | CRC Hypermethylated | 0.282 | 45.708 | Novel |
| cg14037665 | GeneID:7837 | *PXDN* | 2 | 300 | 4.12E-06 | 2.26 | CRC Hypermethylated | 0.282 | 39.174 | Novel |
| cg19064258 | GeneID:9956 | *HS3ST2* | 16 | 257 | 6.65E-07 | 1.95 | CRC Hypermethylated | 0.281 | 42.740 | Previously reported |
| cg24019564 | GeneID:864 | *RUNX3* | 1 | 0 | 2.51E-06 | 2.18 | CRC Hypermethylated | 0.281 | 43.123 | Previously reported |
| cg00489401 | GeneID:2324 | *FLT4* | 5 | 691 | 3.41E-08 | 1.59 | CRC Hypermethylated | 0.281 | 54.689 | Previously reported |
| cg21902544 | GeneID:147381 | *CBLN2* | 18 | 47 | 1.09E-06 | 2.41 | CRC Hypermethylated | 0.281 | 44.046 | Novel |
| cg24625128 | GeneID:83700 | *JAM3* | 11 | 244 | 1.10E-07 | 3.99 | CRC Hypermethylated | 0.281 | 50.804 | Novel |
| cg08832227 | GeneID:3736 | *KCNA1* | 12 | 148 | 5.57E-08 | 1.60 | CRC Hypermethylated | 0.281 | 40.547 | Novel |
| cg04072323 | GeneID:80309 | *SKIP* | 2 | 17 | 5.53E-06 | 2.34 | CRC Hypermethylated | 0.280 | 41.772 | Novel |
| cg22619018 | GeneID:64478 | *CSMD1* | 8 | 296 | 4.84E-06 | 1.58 | CRC Hypermethylated | 0.280 | 36.136 | Novel |
| cg23113963 | GeneID:6338 | *SCNN1B* | 16 | 347 | 7.19E-07 | 2.05 | CRC Hypermethylated | 0.280 | 44.940 | Novel |
| cg23316360 | GeneID:1910 | *EDNRB* | 13 | 0 | 5.60E-08 | 1.56 | CRC Hypermethylated | 0.280 | 52.197 | Novel |
| cg13398291 | GeneID:6422 | *SFRP1* | 8 | 0 | 1.92E-05 | 1.62 | CRC Hypermethylated | 0.280 | 28.211 | Previously reported |
| cg14538332 | GeneID:6383 | *SDC2* | 8 | 298 | 2.92E-06 | 3.08 | CRC Hypermethylated | 0.279 | 44.880 | Novel |
| cg19764436 | GeneID:2781 | *GNAZ* | 22 | 591 | 2.47E-06 | 3.41 | CRC Hypermethylated | 0.278 | 42.143 | Novel |
| cg11747771 | GeneID:2731 | *GLDC* | 9 | 182 | 4.56E-05 | 6.29 | CRC Hypermethylated | 0.278 | 33.725 | Novel |
| cg26035366 | GeneID:116135 | *LRRC3B* | 3 | 321 | 4.48E-06 | 2.48 | CRC Hypermethylated | 0.278 | 36.852 | Previously reported |
| cg25141674 | GeneID:79940 | *C6orf155* | 6 | 241 | 3.93E-06 | 1.81 | CRC Hypermethylated | 0.277 | 38.367 | Novel |
| cg07536847 | GeneID:5081 | *PAX7* | 1 | 268 | 1.36E-07 | 1.85 | CRC Hypermethylated | 0.277 | 49.698 | Novel |
| cg22836229 | GeneID:79645 | *EFCAB1* | 8 | 74 | 1.20E-06 | 1.88 | CRC Hypermethylated | 0.276 | 42.488 | Novel |
| cg13652336 | GeneID:80243 | *DEPDC2* | 8 | 591 | 1.22E-05 | 2.20 | CRC Hypermethylated | 0.275 | 37.201 | Novel |
| cg06151165 | GeneID:30813 | *VSX1* | 20 | 513 | 6.46E-07 | 2.32 | CRC Hypermethylated | 0.275 | 44.418 | Novel |
| cg10332700 | GeneID:55786 | *ZNF415* | 19 | 68 | 0.000193 | 1.97 | CRC Hypermethylated | 0.275 | 26.021 | Novel |
| cg14056644 | GeneID:5308 | *PITX2* | 4 | 597 | 5.23E-06 | 1.95 | CRC Hypermethylated | 0.275 | 44.075 | Novel |
| cg22131691 | GeneID:5137 | *PDE1C* | 7 | 3 | 1.40E-06 | 2.26 | CRC Hypermethylated | 0.274 | 44.364 | Novel |
| cg15045441 | GeneID:79940 | *C6orf155* | 6 | 16 | 5.86E-07 | 3.01 | CRC Hypermethylated | 0.274 | 45.972 | Novel |
| cg07671949 | GeneID:2099 | *ESR1* | 6 | 348 | 1.40E-07 | 1.95 | CRC Hypermethylated | 0.273 | 49.842 | Previously reported |
| cg18221862 | GeneID:23671 | *TMEFF2* | 2 | 414 | 1.90E-05 | 2.60 | CRC Hypermethylated | 0.273 | 34.323 | Previously reported |
| cg14419187 | GeneID:285175 | *C2orf21* | 2 | 377 | 6.78E-06 | 1.69 | CRC Hypermethylated | 0.273 | 32.800 | Novel |
| cg04922810 | GeneID:1395 | *CRHR2* | 7 | 8 | 5.38E-07 | 3.20 | CRC Hypermethylated | 0.273 | 50.015 | Novel |
| cg21376883 | GeneID:88 | *ACTN2* | 1 | 433 | 2.75E-09 | 1.60 | CRC Hypermethylated | 0.272 | 46.700 | Novel |
| cg26747293 | GeneID:133584 | *FLJ39155* | 5 | 137 | 0.000165 | 4.04 | CRC Hypermethylated | 0.272 | 30.219 | Novel |
| cg09099744 | GeneID:1029 | *CDKN2A* | 9 | 0 | 1.42E-05 | 2.93 | CRC Hypermethylated | 0.272 | 32.766 | Previously reported |
| cg13589108 | GeneID:57795 | *FAM5B* | 1 | 47 | 3.71E-05 | 4.84 | CRC Hypermethylated | 0.272 | 36.520 | Novel |
| cg23002761 | GeneID:54751 | *FBLIM1* | 1 | 387 | 4.50E-07 | 1.87 | CRC Hypermethylated | 0.272 | 48.218 | Novel |
| cg07307078 | GeneID:84617 | *TUBB6* | 18 | 627 | 8.80E-05 | 2.72 | CRC Hypermethylated | 0.271 | 35.636 | Novel |
| cg07480567 | GeneID:9955 | *HS3ST3A1* | 17 | 70 | 5.81E-07 | 2.10 | CRC Hypermethylated | 0.271 | 45.968 | Novel |
| cg16098981 | GeneID:79953 | *C20orf39* | 20 | 30 | 7.07E-07 | 2.40 | CRC Hypermethylated | 0.271 | 44.589 | Novel |
| cg06243556 | GeneID:65982 | *ZNF447* | 19 | 104 | 7.15E-05 | 2.81 | CRC Hypermethylated | 0.271 | 33.459 | Novel |
| cg06291867 | GeneID:3363 | *HTR7* | 10 | 509 | 5.56E-06 | 2.08 | CRC Hypermethylated | 0.271 | 40.376 | Novel |
| cg25990647 | GeneID:4986 | *OPRK1* | 8 | 113 | 1.55E-06 | 3.86 | CRC Hypermethylated | 0.271 | 33.099 | Novel |
| cg23857226 | GeneID:79891 | *ZNF671* | 19 | 91 | 1.24E-05 | 2.28 | CRC Hypermethylated | 0.271 | 28.588 | Novel |
| cg01722994 | GeneID:2903 | *GRIN2A* | 16 | 706 | 7.27E-07 | 5.64 | CRC Hypermethylated | 0.270 | 46.669 | Novel |
| cg20498685 | GeneID:7291 | *TWIST1* | 7 | 0 | 4.92E-07 | 3.95 | CRC Hypermethylated | 0.270 | 44.046 | Previously reported |
| cg02932669 | GeneID:259232 | *VGCNL1* | 13 | 448 | 3.08E-05 | 1.85 | CRC Hypermethylated | 0.269 | 31.524 | Novel |
| cg26651233 | GeneID:23768 | *FLRT2* | 14 | 387 | 6.08E-07 | 1.96 | CRC Hypermethylated | 0.269 | 49.064 | Novel |
| cg13168820 | GeneID:11122 | *PTPRT* | 20 | 201 | 8.73E-07 | 2.02 | CRC Hypermethylated | 0.269 | 47.055 | Novel |
| cg25044651 | GeneID:206338 | *FLJ90650* | 5 | 449 | 1.25E-08 | 1.76 | CRC Hypermethylated | 0.268 | 50.048 | Novel |
| cg06490988 | GeneID:2624 | *GATA2* | 3 | 27 | 1.01E-05 | 1.72 | CRC Hypermethylated | 0.268 | 42.202 | Novel |
| cg11004890 | GeneID:83959 | *SLC4A11* | 20 | 127 | 4.43E-07 | 1.86 | CRC Hypermethylated | 0.268 | 47.338 | Novel |
| cg10364513 | GeneID:6258 | *RXRG* | 1 | 51 | 9.11E-06 | 2.47 | CRC Hypermethylated | 0.267 | 39.135 | Novel |
| cg25993718 | GeneID:140689 | *CBLN4* | 20 | 184 | 2.82E-09 | 1.78 | CRC Hypermethylated | 0.267 | 57.930 | Novel |
| cg14659404 | GeneID:460 | *ASTN* | 1 | 5 | 1.20E-05 | 3.04 | CRC Hypermethylated | 0.266 | 39.586 | Novel |
| cg12847373 | GeneID:1910 | *EDNRB* | 13 | 0 | 1.86E-09 | 2.56 | CRC Hypermethylated | 0.266 | 58.558 | Novel |
| cg04490714 | GeneID:6530 | *SLC6A2* | 16 | 8 | 1.15E-07 | 1.63 | CRC Hypermethylated | 0.266 | 49.122 | Novel |
| cg01661993 | GeneID:57348 | *TTYH1* | 19 | 331 | 5.40E-06 | 3.53 | CRC Hypermethylated | 0.265 | 38.725 | Novel |
| cg13265789 | GeneID:8633 | *UNC5C* | 4 | 422 | 2.18E-05 | 2.20 | CRC Hypermethylated | 0.265 | 36.949 | Previously reported |
| cg23710218 | GeneID:9242 | *MSC* | 8 | 33 | 1.18E-07 | 1.59 | CRC Hypermethylated | 0.265 | 56.568 | Novel |
| cg06516124 | GeneID:7490 | *WT1* | 11 | 0 | 8.18E-09 | 2.46 | CRC Hypermethylated | 0.265 | 46.752 | Previously reported |
| cg17923358 | GeneID:5649 | *RELN* | 7 | 344 | 5.76E-05 | 1.92 | CRC Hypermethylated | 0.265 | 31.176 | Novel |
| cg17619823 | GeneID:155 | *ADRB3* | 8 | 709 | 9.81E-05 | 1.74 | CRC Hypermethylated | 0.265 | 30.143 | Novel |
| cg06357925 | GeneID:5800 | *PTPRO* | 12 | 156 | 0.000664 | 3.38 | CRC Hypermethylated | 0.265 | 26.600 | Novel |
| cg09626984 | GeneID:2626 | *GATA4* | 8 | 0 | 1.41E-06 | 1.73 | CRC Hypermethylated | 0.265 | 46.568 | Previously reported |
| cg03289872 | GeneID:63934 | *ZNF667* | 19 | 359 | 1.30E-06 | 2.02 | CRC Hypermethylated | 0.265 | 39.822 | Novel |
| cg12005098 | GeneID:387700 | *SLC16A12* | 10 | 25 | 0.000122 | 1.91 | CRC Hypermethylated | 0.264 | 25.336 | Novel |
| cg22467567 | GeneID:3488 | *IGFBP5* | 2 | 386 | 9.87E-06 | 3.80 | CRC Hypermethylated | 0.264 | 38.417 | Novel |
| cg20339230 | GeneID:8128 | *ST8SIA2* | 15 | 220 | 0.00011 | 4.14 | CRC Hypermethylated | 0.263 | 32.100 | Novel |
| cg10743104 | GeneID:10630 | *PDPN* | 1 | 46 | 3.75E-06 | 2.23 | CRC Hypermethylated | 0.263 | 37.904 | Novel |
| cg14859460 | GeneID:2916 | *GRM6* | 5 | 120 | 4.35E-08 | 1.52 | CRC Hypermethylated | 0.263 | 46.004 | Novel |
| cg19326876 | GeneID:57194 | *ATP10A* | 15 | 0 | 3.24E-05 | 2.09 | CRC Hypermethylated | 0.263 | 35.172 | Novel |
| cg17471928 | GeneID:342667 | *STAC2* | 17 | 144 | 2.02E-06 | 2.75 | CRC Hypermethylated | 0.263 | 38.984 | Novel |
| cg19896198 | GeneID:943 | *TNFRSF8* | 1 | 172 | 1.75E-05 | 2.69 | CRC Hypermethylated | 0.262 | 33.852 | Novel |
| cg02164046 | GeneID:6750 | *SST* | 3 | 53 | 1.94E-07 | 1.65 | CRC Hypermethylated | 0.262 | 46.791 | Novel |
| cg03616357 | GeneID:79884 | *FLJ21159* | 4 | 180 | 8.43E-05 | 4.32 | CRC Hypermethylated | 0.262 | 31.965 | Novel |
| cg19988449 | GeneID:646 | *BNC1* | 15 | 660 | 5.51E-07 | 1.71 | CRC Hypermethylated | 0.262 | 42.153 | Previously reported |
| cg16517394 | GeneID:7292 | *TNFSF4* | 1 | 109 | 4.24E-06 | 2.47 | CRC Hypermethylated | 0.262 | 49.275 | Novel |
| cg18399321 | GeneID:4248 | *MGAT3* | 22 | 265 | 2.12E-05 | 6.93 | CRC Hypermethylated | 0.262 | 31.835 | Novel |
| cg24715245 | GeneID:7345 | *UCHL1* | 4 | 135 | 7.18E-06 | 2.47 | CRC Hypermethylated | 0.262 | 41.013 | Novel |
| cg23642747 | GeneID:9118 | *INA* | 10 | 275 | 1.97E-07 | 2.54 | CRC Hypermethylated | 0.261 | 49.029 | Novel |
| cg05222924 | GeneID:7490 | *WT1* | 11 | 0 | 1.04E-06 | 2.18 | CRC Hypermethylated | 0.261 | 42.055 | Previously reported |
| cg06722633 | GeneID:2899 | *GRIK3* | 1 | 535 | 2.93E-06 | 1.62 | CRC Hypermethylated | 0.261 | 39.820 | Novel |
| cg12006284 | GeneID:7490 | *WT1* | 11 | 0 | 2.63E-08 | 1.71 | CRC Hypermethylated | 0.261 | 44.583 | Previously reported |
| cg10730712 | GeneID:91522 | *COL23A1* | 5 | 271 | 0.000952 | 1.92 | CRC Hypermethylated | 0.261 | 27.518 | Novel |
| cg07846220 | GeneID:284217 | *LAMA1* | 18 | 133 | 2.62E-08 | 1.51 | CRC Hypermethylated | 0.260 | 46.085 | Novel |
| cg22392666 | GeneID:53822 | *FXYD7* | 19 | 13 | 1.43E-05 | 2.51 | CRC Hypermethylated | 0.260 | 38.198 | Novel |
| cg14409941 | GeneID:9509 | *ADAMTS2* | 5 | 548 | 3.28E-05 | 2.93 | CRC Hypermethylated | 0.260 | 33.402 | Novel |
| cg00687686 | GeneID:65009 | *NDRG4* | 16 | 381 | 0.000126 | 9.34 | CRC Hypermethylated | 0.260 | 29.425 | Previously reported |
| cg24924779 | GeneID:3755 | *KCNG1* | 20 | 381 | 7.26E-06 | 2.37 | CRC Hypermethylated | 0.260 | 34.339 | Novel |
| cg20113732 | GeneID:4745 | *NELL1* | 11 | 44 | 5.48E-06 | 2.14 | CRC Hypermethylated | 0.260 | 37.752 | Novel |
| cg18482268 | GeneID:5459 | *POU4F3* | 5 | 289 | 6.41E-07 | 2.11 | CRC Hypermethylated | 0.259 | 43.261 | Novel |
| cg15836660 | GeneID:1910 | *EDNRB* | 13 | 0 | 3.06E-07 | 2.29 | CRC Hypermethylated | 0.259 | 47.487 | Novel |
| cg07054095 | GeneID:256051 | *ZNF549* | 19 | 163 | 3.13E-05 | 3.64 | CRC Hypermethylated | 0.259 | 34.345 | Novel |
| cg03943081 | GeneID:256536 | *TCERG1L* | 10 | 660 | 1.18E-07 | 2.39 | CRC Hypermethylated | 0.258 | 42.812 | Novel |
| cg15183083 | GeneID:3739 | *KCNA4* | 11 | 178 | 1.42E-05 | 2.03 | CRC Hypermethylated | 0.258 | 34.302 | Novel |
| cg04278702 | GeneID:3354 | *HTR1E* | 6 | 11 | 3.58E-05 | 2.42 | CRC Hypermethylated | 0.258 | 33.069 | Novel |
| cg23129478 | GeneID:29906 | *ST8SIA5* | 18 | 883 | 6.59E-06 | 5.83 | CRC Hypermethylated | 0.257 | 38.757 | Novel |
| cg05396987 | GeneID:132332 | *FLJ30834* | 4 | 820 | 1.17E-07 | 1.92 | CRC Hypermethylated | 0.257 | 45.297 | Novel |
| cg23097006 | GeneID:30813 | *VSX1* | 20 | 1050 | 0.000161 | 2.17 | CRC Hypermethylated | 0.257 | 27.822 | Novel |
| cg13462129 | GeneID:1749 | *DLX5* | 7 | 0 | 1.79E-06 | 1.70 | CRC Hypermethylated | 0.257 | 39.806 | Novel |
| cg02217159 | GeneID:202559 | *KHDRBS2* | 6 | 565 | 1.98E-06 | 2.49 | CRC Hypermethylated | 0.257 | 41.259 | Novel |
| cg27637521 | GeneID:9021 | *SOCS3* | 17 | 956 | 0.000265 | 3.81 | CRC Hypermethylated | 0.256 | 27.198 | Novel |
| cg27351358 | GeneID:627 | *BDNF* | 11 | 38 | 3.56E-06 | 3.17 | CRC Hypermethylated | 0.256 | 40.197 | Previously reported |
| cg15717808 | GeneID:56479 | *KCNQ5* | 6 | 183 | 2.11E-05 | 4.03 | CRC Hypermethylated | 0.256 | 36.717 | Novel |
| cg18722841 | GeneID:401 | *PHOX2A* | 11 | 222 | 1.28E-06 | 2.03 | CRC Hypermethylated | 0.256 | 43.653 | Novel |
| cg19461644 | GeneID:1306 | *COL15A1* | 9 | 427 | 2.38E-06 | 1.91 | CRC Hypermethylated | 0.255 | 42.964 | Novel |
| cg21972382 | GeneID:79745 | *RSNL2* | 2 | 758 | 1.12E-05 | 2.25 | CRC Hypermethylated | 0.255 | 37.560 | Novel |
| cg00654814 | GeneID:146664 | *MGAT5B* | 17 | 270 | 0.000104 | 2.27 | CRC Hypermethylated | 0.255 | 30.309 | Novel |
| cg04551925 | GeneID:358 | *AQP1* | 7 | 272 | 4.09E-08 | 1.47 | CRC Hypermethylated | 0.255 | 55.312 | Novel |
| cg19629292 | GeneID:55079 | *ZNF312* | 3 | 41 | 1.92E-05 | 2.47 | CRC Hypermethylated | 0.255 | 36.223 | Novel |
| cg10303487 | GeneID:1807 | *DPYS* | 8 | 219 | 4.72E-05 | 1.56 | CRC Hypermethylated | 0.254 | 27.745 | Novel |
| cg05368341 | GeneID:148281 | *SYT6* | 1 | 7 | 2.28E-05 | 3.23 | CRC Hypermethylated | 0.254 | 34.290 | Novel |
| cg19018097 | GeneID:254122 | *FLJ30934* | 11 | 82 | 9.89E-06 | 4.74 | CRC Hypermethylated | 0.254 | 36.238 | Novel |
| cg03775422 | GeneID:222008 | *MGC33530* | 7 | 51 | 1.22E-08 | 2.42 | CRC Hypermethylated | 0.254 | 55.249 | Novel |
| cg11732619 | GeneID:6586 | *SLIT3* | 5 | 100 | 2.78E-05 | 1.84 | CRC Hypermethylated | 0.254 | 32.857 | Novel |
| cg24840099 | GeneID:4487 | *MSX1* | 4 | 0 | 6.39E-07 | 1.57 | CRC Hypermethylated | 0.254 | 44.104 | Previously reported |
| cg13262687 | GeneID:5458 | *POU4F2* | 4 | 509 | 7.95E-07 | 1.88 | CRC Hypermethylated | 0.254 | 44.426 | Novel |
| cg25856383 | GeneID:1545 | *CYP1B1* | 2 | 73 | 7.07E-05 | 5.28 | CRC Hypermethylated | 0.253 | 34.268 | Previously reported |
| cg24745738 | GeneID:1910 | *EDNRB* | 13 | 0 | 1.64E-05 | 2.27 | CRC Hypermethylated | 0.253 | 34.095 | Novel |
| cg17456704 | GeneID:90589 | *ZNF625* | 19 | 247 | 2.10E-05 | 2.59 | CRC Hypermethylated | 0.253 | 33.209 | Novel |
| cg01988129 | GeneID:137872 | *ADHFE1* | 8 | 202 | 9.98E-08 | 1.48 | CRC Hypermethylated | 0.253 | 52.565 | Novel |
| cg06856528 | GeneID:23671 | *TMEFF2* | 2 | 239 | 4.05E-06 | 2.21 | CRC Hypermethylated | 0.253 | 40.823 | Previously reported |
| cg07748540 | GeneID:80310 | *PDGFD* | 11 | 270 | 9.01E-05 | 5.28 | CRC Hypermethylated | 0.253 | 30.669 | Novel |
| cg27352992 | GeneID:1272 | *CNTN1* | 12 | 734 | 4.12E-07 | 2.44 | CRC Hypermethylated | 0.252 | 48.016 | Novel |
| cg19713460 | GeneID:9145 | *SYNGR1* | 22 | 424 | 2.45E-07 | 1.56 | CRC Hypermethylated | 0.252 | 49.564 | Novel |
| cg17886204 | GeneID:196968 | *DKFZp434I1020* | 15 | 138 | 0.000453 | 5.40 | CRC Hypermethylated | 0.252 | 22.265 | Novel |
| cg19591881 | GeneID:947 | *CD34* | 1 | 43 | 2.75E-06 | 1.89 | CRC Hypermethylated | 0.252 | 36.597 | Previously reported |
| cg10300684 | GeneID:2290 | *FOXG1B* | 14 | 36 | 2.20E-07 | 1.84 | CRC Hypermethylated | 0.252 | 47.444 | Novel |
| cg26620959 | GeneID:23345 | *SYNE1* | 6 | 45 | 1.60E-05 | 2.91 | CRC Hypermethylated | 0.251 | 36.158 | Novel |
| cg22709192 | GeneID:3227 | *HOXC11* | 12 | 20 | 4.90E-07 | 1.49 | CRC Hypermethylated | 0.251 | 35.124 | Novel |
| cg23587449 | GeneID:9227 | *LRAT* | 4 | 1474 | 4.76E-07 | 2.38 | CRC Hypermethylated | 0.251 | 43.536 | Novel |
| cg13234863 | GeneID:121256 | *KIAA1944* | 12 | 925 | 3.83E-07 | 1.63 | CRC Hypermethylated | 0.251 | 42.981 | Novel |
| cg14070647 | GeneID:340419 | *RSPO2* | 8 | 302 | 2.37E-05 | 2.65 | CRC Hypermethylated | 0.251 | 36.329 | Novel |
| cg12539975 | GeneID:59335 | *PRDM12* | 9 | 398 | 0.000263 | 3.37 | CRC Hypermethylated | 0.251 | 31.559 | Novel |
| cg16792800 | GeneID:10082 | *GPC6* | 13 | 266 | 6.54E-06 | 1.55 | CRC Hypermethylated | 0.251 | 42.011 | Novel |
| cg01546563 | GeneID:2626 | *GATA4* | 8 | 0 | 3.09E-08 | 2.21 | CRC Hypermethylated | 0.251 | 48.357 | Previously reported |
| cg04270799 | GeneID:3798 | *KIF5A* | 12 | 212 | 8.68E-06 | 3.65 | CRC Hypermethylated | 0.251 | 39.187 | Novel |
| cg03238797 | GeneID:170692 | *ADAMTS18* | 16 | 118 | 2.15E-05 | 2.03 | CRC Hypermethylated | 0.250 | 32.523 | Novel |
| cg07379574 | GeneID:25789 | *C19orf4* | 19 | 461 | 2.05E-05 | 2.16 | CRC Hypermethylated | 0.250 | 34.926 | Novel |
| cg20052718 | GeneID:7291 | *TWIST1* | 7 | 0 | 9.61E-06 | 3.74 | CRC Hypermethylated | 0.250 | 39.347 | Previously reported |
| cg12699371 | GeneID:2587 | *GALR1* | 18 | 309 | 1.60E-05 | 2.31 | CRC Hypermethylated | 0.249 | 33.398 | Previously reported |
| cg19055231 | GeneID:6769 | *STAC* | 3 | 300 | 7.61E-06 | 2.14 | CRC Hypermethylated | 0.249 | 34.795 | Novel |
| cg20890210 | GeneID:9312 | *KCNB2* | 8 | 555 | 1.69E-07 | 1.77 | CRC Hypermethylated | 0.249 | 49.686 | Novel |
| cg25938646 | GeneID:114798 | *SLITRK1* | 13 | 153 | 2.09E-05 | 1.84 | CRC Hypermethylated | 0.249 | 31.343 | Novel |
| cg04765277 | GeneID:399717 | *FLJ45983* | 10 | 768 | 1.26E-06 | 2.32 | CRC Hypermethylated | 0.248 | 39.772 | Novel |
| cg17453778 | GeneID:846 | *CASR* | 3 | 31 | 1.65E-06 | 1.96 | CRC Hypermethylated | 0.248 | 46.443 | Novel |
| cg03469054 | GeneID:121256 | *KIAA1944* | 12 | 352 | 1.25E-06 | 1.73 | CRC Hypermethylated | 0.248 | 42.022 | Novel |
| cg20080624 | GeneID:1749 | *DLX5* | 7 | 0 | 1.13E-05 | 2.07 | CRC Hypermethylated | 0.248 | 40.580 | Novel |
| cg02245378 | GeneID:151278 | *FLJ32447* | 2 | 1095 | 1.06E-05 | 1.73 | CRC Hypermethylated | 0.248 | 38.598 | Novel |
| cg06201642 | GeneID:81849 | *ST6GALNAC5* | 1 | 12 | 1.48E-07 | 3.08 | CRC Hypermethylated | 0.248 | 49.562 | Novel |
| cg22341310 | GeneID:84215 | *ZNF541* | 19 | 429 | 6.54E-06 | 1.61 | CRC Hypermethylated | 0.248 | 37.224 | Novel |
| cg25047001 | GeneID:2903 | *GRIN2A* | 16 | 406 | 1.79E-07 | 4.35 | CRC Hypermethylated | 0.247 | 50.279 | Novel |
| cg05839235 | GeneID:4883 | *NPR3* | 5 | 572 | 2.58E-07 | 1.91 | CRC Hypermethylated | 0.247 | 56.269 | Novel |
| cg26043257 | GeneID:952 | *CD38* | 4 | 307 | 5.83E-06 | 2.30 | CRC Hypermethylated | 0.247 | 33.431 | Novel |
| cg23300372 | GeneID:9607 | *CART* | 5 | 438 | 1.90E-07 | 1.86 | CRC Hypermethylated | 0.247 | 40.223 | Novel |
| cg22040627 | GeneID:284111 | *SLC13A5* | 17 | 366 | 4.05E-06 | 2.40 | CRC Hypermethylated | 0.247 | 45.446 | Novel |
| cg02569613 | GeneID:196740 | *C10orf72* | 10 | 432 | 7.39E-05 | 1.92 | CRC Hypermethylated | 0.247 | 29.826 | Novel |
| cg13791131 | GeneID:51214 | *IGF2AS* | 11 | 145 | 1.34E-05 | 1.72 | CRC Hypermethylated | 0.247 | 41.373 | Previously reported |
| cg00243313 | GeneID:50805 | *IRX4* | 5 | 533 | 6.18E-06 | 2.81 | CRC Hypermethylated | 0.247 | 38.109 | Novel |
| cg26963271 | GeneID:5142 | *PDE4B* | 1 | 217 | 2.07E-06 | 2.41 | CRC Hypermethylated | 0.247 | 44.139 | Novel |
| cg10398682 | GeneID:646 | *BNC1* | 15 | 0 | 4.11E-06 | 1.66 | CRC Hypermethylated | 0.247 | 43.031 | Previously reported |
| cg17194182 | GeneID:2056 | *EPO* | 7 | 269 | 0.000949 | 6.09 | CRC Hypermethylated | 0.247 | 24.716 | Previously reported |
| cg14262937 | GeneID:4988 | *OPRM1* | 6 | 92 | 7.57E-06 | 1.97 | CRC Hypermethylated | 0.246 | 37.379 | Novel |
| cg00472814 | GeneID:9510 | *ADAMTS1* | 21 | 52 | 3.99E-05 | 2.28 | CRC Hypermethylated | 0.246 | 34.004 | Novel |
| cg07036530 | GeneID:2849 | *GPR26* | 10 | 609 | 8.02E-06 | 1.68 | CRC Hypermethylated | 0.246 | 36.060 | Novel |
| cg21264055 | GeneID:80742 | *PRR3* | 6 | 1421 | 5.74E-05 | 2.87 | CRC Hypermethylated | 0.245 | 36.811 | Novel |
| cg18877506 | GeneID:10630 | *PDPN* | 1 | 303 | 6.38E-05 | 2.34 | CRC Hypermethylated | 0.245 | 28.844 | Novel |
| cg07186138 | GeneID:27350 | *APOBEC3C* | 22 | 131 | 7.08E-06 | 2.62 | CRC Hypermethylated | 0.245 | 35.469 | Novel |
| cg12238343 | GeneID:51289 | *RLN3R1* | 5 | 87 | 3.03E-07 | 3.17 | CRC Hypermethylated | 0.245 | 52.313 | Novel |
| cg16714091 | GeneID:92949 | *ADAMTSL1* | 9 | 265 | 1.65E-06 | 2.34 | CRC Hypermethylated | 0.245 | 51.338 | Novel |
| cg00792849 | GeneID:57369 | *CX36* | 15 | 79 | 8.69E-07 | 1.98 | CRC Hypermethylated | 0.245 | 40.192 | Novel |
| cg13266631 | GeneID:4747 | *NEFL* | 8 | 977 | 2.94E-05 | 1.81 | CRC Hypermethylated | 0.244 | 30.993 | Previously reported |
| cg20645065 | GeneID:249 | *ALPL* | 1 | 269 | 0.00044 | 2.21 | CRC Hypermethylated | 0.244 | 29.377 | Novel |
| cg27223047 | GeneID:2201 | *FBN2* | 5 | 1090 | 9.76E-09 | 1.64 | CRC Hypermethylated | 0.244 | 53.788 | Novel |
| cg18236477 | GeneID:51761 | *ATP8A2* | 13 | 49 | 1.32E-07 | 1.54 | CRC Hypermethylated | 0.244 | 51.554 | Novel |
| cg12109455 | GeneID:10570 | *DPYSL4* | 10 | 333 | 5.82E-07 | 1.75 | CRC Hypermethylated | 0.243 | 50.197 | Novel |
| cg19965810 | GeneID:90134 | *KCNH7* | 2 | 553 | 9.26E-07 | 1.69 | CRC Hypermethylated | 0.243 | 39.199 | Novel |
| cg06812977 | GeneID:55328 | *C10orf59* | 10 | 28 | 0.000587 | 3.12 | CRC Hypermethylated | 0.243 | 24.014 | Novel |
| cg14696396 | GeneID:53346 | *TM6SF1* | 15 | 66 | 1.22E-08 | 1.70 | CRC Hypermethylated | 0.243 | 54.675 | Novel |
| cg11323198 | GeneID:1006 | *CDH8* | 16 | 230 | 4.63E-06 | 1.46 | CRC Hypermethylated | 0.242 | 37.613 | Novel |
| cg08118311 | GeneID:27164 | *SALL3* | 18 | 126 | 8.21E-07 | 1.76 | CRC Hypermethylated | 0.242 | 39.475 | Novel |
| cg14323109 | GeneID:3791 | *KDR* | 4 | 181 | 2.55E-05 | 2.08 | CRC Hypermethylated | 0.241 | 31.256 | Previously reported |
| cg23496260 | GeneID:10409 | *BASP1* | 5 | 339 | 7.48E-06 | 6.81 | CRC Hypermethylated | 0.241 | 41.145 | Novel |
| cg05825950 | GeneID:1378 | *CR1* | 1 | 74 | 2.92E-05 | 2.82 | CRC Hypermethylated | 0.241 | 32.662 | Novel |
| cg20491707 | GeneID:5540 | *PPYR1* | 10 | 251 | 2.80E-06 | 1.51 | CRC Hypermethylated | 0.241 | 42.586 | Novel |
| cg00030047 | GeneID:148646 | *C1orf188* | 1 | 130 | 0.000269 | 1.49 | CRC Hypermethylated | 0.241 | 33.175 | Novel |
| cg12614105 | GeneID:4852 | *NPY* | 7 | 626 | 1.06E-06 | 1.71 | CRC Hypermethylated | 0.240 | 38.852 | Previously reported |
| cg00970325 | GeneID:344838 | *PAQR9* | 3 | 998 | 0.000174 | 3.41 | CRC Hypermethylated | 0.240 | 30.002 | Novel |
| cg06277657 | GeneID:9162 | *DGKI* | 7 | 765 | 5.49E-07 | 1.55 | CRC Hypermethylated | 0.240 | 42.828 | Novel |
| cg06269753 | GeneID:9242 | *MSC* | 8 | 752 | 6.31E-07 | 2.35 | CRC Hypermethylated | 0.239 | 44.817 | Novel |
| cg03630088 | GeneID:119587 | *CPXM2* | 10 | 26 | 2.18E-05 | 2.33 | CRC Hypermethylated | 0.239 | 38.691 | Novel |
| cg20312228 | GeneID:348807 | *CCDC37* | 3 | 75 | 5.31E-06 | 1.64 | CRC Hypermethylated | 0.239 | 44.575 | Novel |
| cg16092786 | GeneID:7490 | *WT1* | 11 | 0 | 6.20E-05 | 2.40 | CRC Hypermethylated | 0.239 | 31.791 | Previously reported |
| cg16670497 | GeneID:2946 | *GSTM2* | 1 | 222 | 2.08E-06 | 4.13 | CRC Hypermethylated | 0.239 | 46.499 | Previously reported |
| cg21688264 | GeneID:9892 | *SNAP91* | 6 | 52 | 0.00014 | 2.25 | CRC Hypermethylated | 0.238 | 30.496 | Novel |
| cg09147222 | GeneID:131034 | *CPNE4* | 3 | 282 | 0.000146 | 3.30 | CRC Hypermethylated | 0.238 | 26.151 | Novel |
| cg21907579 | GeneID:6910 | *TBX5* | 12 | 379 | 8.66E-07 | 1.81 | CRC Hypermethylated | 0.238 | 38.719 | Novel |
| cg15489294 | GeneID:206338 | *FLJ90650* | 5 | 418 | 4.11E-05 | 1.79 | CRC Hypermethylated | 0.238 | 35.291 | Novel |
| cg01519742 | GeneID:152789 | *JAKMIP1* | 4 | 191 | 0.000599 | 4.24 | CRC Hypermethylated | 0.238 | 24.743 | Novel |
| cg13549845 | GeneID:2895 | *GRID2* | 4 | 152 | 3.03E-05 | 2.03 | CRC Hypermethylated | 0.237 | 29.547 | Novel |
| cg23092823 | GeneID:127435 | *PODN* | 1 | 727 | 3.94E-06 | 2.75 | CRC Hypermethylated | 0.237 | 44.036 | Novel |
| cg13921352 | GeneID:151647 | *FAM19A4* | 3 | 179 | 0.000178 | 2.05 | CRC Hypermethylated | 0.237 | 27.241 | Novel |
| cg20404387 | GeneID:163933 | *FAM43B* | 1 | 364 | 4.71E-07 | 1.82 | CRC Hypermethylated | 0.237 | 42.849 | Novel |
| cg20308679 | GeneID:2487 | *FRZB* | 2 | 91 | 4.72E-05 | 2.19 | CRC Hypermethylated | 0.237 | 27.119 | Novel |
| cg18488855 | GeneID:4857 | *NOVA1* | 14 | 326 | 0.000103 | 2.88 | CRC Hypermethylated | 0.237 | 31.179 | Novel |
| cg15690721 | GeneID:7639 | *ZNF85* | 19 | 78 | 6.38E-05 | 3.78 | CRC Hypermethylated | 0.236 | 32.462 | Novel |
| cg16501028 | GeneID:7490 | *WT1* | 11 | 0 | 2.70E-05 | 1.48 | CRC Hypermethylated | 0.236 | 40.960 | Previously reported |
| cg22674717 | GeneID:6299 | *SALL1* | 16 | 242 | 3.51E-05 | 2.13 | CRC Hypermethylated | 0.236 | 32.388 | Novel |
| cg24834740 | GeneID:26051 | *PPP1R16B* | 20 | 204 | 6.34E-05 | 4.23 | CRC Hypermethylated | 0.236 | 31.945 | Novel |
| cg02497758 | GeneID:9935 | *MAFB* | 20 | 682 | 9.84E-05 | 1.61 | CRC Hypermethylated | 0.236 | 31.145 | Novel |
| cg15817236 | GeneID:60529 | *ALX4* | 11 | 354 | 0.000203 | 8.03 | CRC Hypermethylated | 0.236 | 28.870 | Previously reported |
| cg21237591 | GeneID:51214 | *IGF2AS* | 11 | 763 | 3.19E-05 | 1.77 | CRC Hypermethylated | 0.236 | 36.844 | Previously reported |
| cg21790626 | GeneID:7710 | *ZNF154* | 19 | 68 | 7.56E-08 | 1.50 | CRC Hypermethylated | 0.235 | 37.152 | Novel |
| cg18420965 | GeneID:2044 | *EPHA5* | 4 | 364 | 8.82E-06 | 1.55 | CRC Hypermethylated | 0.235 | 30.928 | Previously reported |
| cg05389335 | GeneID:6870 | *TACR3* | 4 | 346 | 1.98E-06 | 1.83 | CRC Hypermethylated | 0.235 | 44.171 | Novel |
| cg17560332 | GeneID:66037 | *BOLL* | 2 | 365 | 1.34E-05 | 2.26 | CRC Hypermethylated | 0.235 | 37.009 | Novel |
| cg20366906 | GeneID:5100 | *PCDH8* | 13 | 393 | 6.48E-07 | 1.62 | CRC Hypermethylated | 0.234 | 44.509 | Novel |
| cg15839448 | GeneID:6422 | *SFRP1* | 8 | 450 | 1.45E-07 | 1.88 | CRC Hypermethylated | 0.234 | 48.181 | Previously reported |
| cg16902509 | GeneID:8516 | *ITGA8* | 10 | 458 | 1.63E-07 | 1.96 | CRC Hypermethylated | 0.233 | 49.912 | Novel |
| cg11038843 | GeneID:27087 | *B3GAT1* | 11 | 421 | 0.000666 | 2.08 | CRC Hypermethylated | 0.233 | 28.378 | Novel |
| cg01839464 | GeneID:1630 | *DCC* | 18 | 0 | 7.73E-05 | 1.53 | CRC Hypermethylated | 0.233 | 30.810 | Previously reported |
| cg03425110 | GeneID:50674 | *NEUROG3* | 10 | 85 | 2.59E-05 | 2.71 | CRC Hypermethylated | 0.233 | 34.073 | Novel |
| cg03495868 | GeneID:5737 | *PTGFR* | 1 | 177 | 1.35E-06 | 2.06 | CRC Hypermethylated | 0.232 | 45.086 | Novel |
| cg12153542 | GeneID:28954 | *REM1* | 20 | 24 | 2.72E-05 | 2.61 | CRC Hypermethylated | 0.232 | 35.753 | Novel |
| cg22461835 | GeneID:148 | *ADRA1A* | 8 | 443 | 7.77E-06 | 2.17 | CRC Hypermethylated | 0.232 | 41.867 | Novel |
| cg11611600 | GeneID:57188 | *ADAMTSL3* | 15 | 316 | 0.000162 | 1.82 | CRC Hypermethylated | 0.232 | 27.840 | Novel |
| cg17256157 | GeneID:4829 | *NMBR* | 6 | 164 | 5.91E-06 | 2.31 | CRC Hypermethylated | 0.232 | 37.659 | Novel |
| cg25875213 | GeneID:163115 | *FLJ37549* | 19 | 97 | 0.00017 | 3.24 | CRC Hypermethylated | 0.232 | 29.472 | Novel |
| cg21250296 | GeneID:3018 | *HIST1H2BB* | 6 | 520 | 6.24E-05 | 3.83 | CRC Hypermethylated | 0.231 | 31.951 | Novel |
| cg04096767 | GeneID:7490 | *WT1* | 11 | 0 | 6.95E-07 | 1.59 | CRC Hypermethylated | 0.231 | 37.191 | Previously reported |
| cg24719984 | GeneID:8499 | *PPFIA2* | 12 | 355 | 1.19E-08 | 1.88 | CRC Hypermethylated | 0.231 | 51.203 | Novel |
| cg10764357 | GeneID:586 | *BCAT1* | 12 | 160 | 8.53E-07 | 2.90 | CRC Hypermethylated | 0.231 | 41.991 | Novel |
| cg10872212 | GeneID:200942 | *KLHDC8B* | 3 | 92 | 7.03E-05 | 4.10 | CRC Hypermethylated | 0.230 | 31.456 | Novel |
| cg25886284 | GeneID:284406 | *ZNF545* | 19 | 132 | 4.09E-05 | 2.68 | CRC Hypermethylated | 0.230 | 28.336 | Novel |
| cg12024292 | GeneID:23245 | *ASTN2* | 9 | 662 | 1.51E-06 | 2.75 | CRC Hypermethylated | 0.230 | 42.480 | Novel |
| cg12874092 | GeneID:7431 | *VIM* | 10 | 221 | 0.000239 | 24.27 | CRC Hypermethylated | 0.230 | 29.793 | Previously reported |
| cg11822659 | GeneID:55816 | *DOK5* | 20 | 524 | 9.08E-07 | 2.00 | CRC Hypermethylated | 0.230 | 39.529 | Novel |
| cg22609576 | GeneID:23111 | *SPG20* | 13 | 209 | 3.64E-05 | 4.87 | CRC Hypermethylated | 0.230 | 35.298 | Novel |
| cg12300353 | GeneID:386617 | *KCTD8* | 4 | 466 | 0.000513 | 1.92 | CRC Hypermethylated | 0.230 | 26.507 | Novel |
| cg26024843 | GeneID:1289 | *COL5A1* | 9 | 297 | 1.41E-06 | 1.80 | CRC Hypermethylated | 0.230 | 42.689 | Novel |
| cg22187630 | GeneID:773 | *CACNA1A* | 19 | 446 | 2.80E-08 | 1.72 | CRC Hypermethylated | 0.230 | 52.189 | Novel |
| cg18110483 | GeneID:7060 | *THBS4* | 5 | 32 | 4.12E-06 | 2.32 | CRC Hypermethylated | 0.229 | 40.497 | Novel |
| cg13282837 | GeneID:8115 | *TCL1A* | 14 | 200 | 7.01E-08 | 1.45 | CRC Hypermethylated | 0.229 | 47.268 | Novel |
| cg24199834 | GeneID:5458 | *POU4F2* | 4 | 38 | 2.29E-05 | 1.58 | CRC Hypermethylated | 0.228 | 33.984 | Novel |
| cg07494047 | GeneID:57343 | *ZNF304* | 19 | 38 | 0.000101 | 3.22 | CRC Hypermethylated | 0.228 | 27.848 | Novel |
| cg06821993 | GeneID:114788 | *CSMD3* | 8 | 231 | 6.20E-07 | 2.44 | CRC Hypermethylated | 0.228 | 47.223 | Novel |
| cg16924616 | GeneID:1749 | *DLX5* | 7 | 526 | 0.000268 | 1.59 | CRC Hypermethylated | 0.228 | 23.893 | Novel |
| cg23239396 | GeneID:799 | *CALCR* | 7 | 55 | 9.34E-06 | 2.13 | CRC Hypermethylated | 0.228 | 39.210 | Novel |
| cg10158080 | GeneID:6660 | *SOX5* | 12 | 484 | 0.000425 | 3.09 | CRC Hypermethylated | 0.227 | 25.285 | Novel |
| cg17177660 | GeneID:7424 | *VEGFC* | 4 | 561 | 2.95E-05 | 1.64 | CRC Hypermethylated | 0.227 | 36.243 | Novel |
| cg23239039 | GeneID:27065 | *D4S234E* | 4 | 363 | 7.83E-07 | 2.49 | CRC Hypermethylated | 0.227 | 44.463 | Novel |
| cg06564900 | GeneID:55137 | *FIGN* | 2 | 55 | 6.28E-06 | 1.84 | CRC Hypermethylated | 0.226 | 39.949 | Novel |
| cg00806490 | GeneID:1012 | *CDH13* | 16 | 295 | 0.001033 | 1.74 | CRC Hypermethylated | 0.225 | 24.399 | Previously reported |
| cg06668300 | GeneID:4118 | *MAL* | 2 | 276 | 4.82E-05 | 4.44 | CRC Hypermethylated | 0.225 | 35.242 | Previously reported |
| cg18888520 | GeneID:65982 | *ZNF447* | 19 | 229 | 0.000165 | 2.57 | CRC Hypermethylated | 0.224 | 28.584 | Novel |
| cg19355190 | GeneID:1959 | *EGR2* | 10 | 329 | 8.60E-05 | 4.82 | CRC Hypermethylated | 0.224 | 35.349 | Novel |
| cg13991233 | GeneID:8522 | *GAS7* | 17 | 690 | 1.09E-06 | 2.95 | CRC Hypermethylated | 0.224 | 44.287 | Previously reported |
| cg06971096 | GeneID:5798 | *PTPRN* | 2 | 552 | 7.92E-05 | 1.60 | CRC Hypermethylated | 0.224 | 30.013 | Novel |
| cg17897879 | GeneID:943 | *TNFRSF8* | 1 | 3 | 0.000304 | 3.77 | CRC Hypermethylated | 0.224 | 30.967 | Novel |
| cg03294619 | GeneID:1482 | *NKX2-5* | 5 | 459 | 0.000154 | 5.21 | CRC Hypermethylated | 0.224 | 30.692 | Novel |
| cg01830294 | GeneID:7472 | *WNT2* | 7 | 149 | 3.63E-05 | 1.49 | CRC Hypermethylated | 0.223 | 33.726 | Previously reported |
| cg08003150 | GeneID:3785 | *KCNQ2* | 20 | 366 | 0.00015 | 3.61 | CRC Hypermethylated | 0.222 | 29.262 | Novel |
| cg07636178 | GeneID:8352 | *HIST1H3C* | 6 | 105 | 0.000538 | 4.07 | CRC Hypermethylated | 0.222 | 24.838 | Novel |
| cg13663218 | GeneID:29953 | *TRHDE* | 12 | 513 | 1.76E-07 | 1.53 | CRC Hypermethylated | 0.221 | 51.513 | Novel |
| cg27016494 | GeneID:1749 | *DLX5* | 7 | 0 | 4.62E-09 | 1.86 | CRC Hypermethylated | 0.221 | 51.593 | Novel |
| cg17793621 | GeneID:57194 | *ATP10A* | 15 | 0 | 0.000252 | 2.11 | CRC Hypermethylated | 0.220 | 35.539 | Novel |
| cg13929328 | GeneID:399823 | *FLJ46831* | 10 | 54 | 5.81E-06 | 1.58 | CRC Hypermethylated | 0.220 | 40.480 | Novel |
| cg16232126 | GeneID:60482 | *SLC5A7* | 2 | 10 | 1.04E-05 | 1.55 | CRC Hypermethylated | 0.219 | 36.784 | Novel |
| cg21238818 | GeneID:89792 | *GAL3ST3* | 11 | 9 | 0.000335 | 1.47 | CRC Hypermethylated | 0.219 | 31.674 | Novel |
| cg19664945 | GeneID:10936 | *GPR75* | 2 | 275 | 1.26E-07 | 1.43 | CRC Hypermethylated | 0.219 | 50.915 | Novel |
| cg18533225 | GeneID:113730 | *KLHDC7B* | 22 | 351 | 3.17E-07 | 1.40 | CRC Hypermethylated | 0.218 | 50.993 | Novel |
| cg16652063 | GeneID:284111 | *SLC13A5* | 17 | 11 | 2.02E-05 | 1.50 | CRC Hypermethylated | 0.218 | 30.760 | Novel |
| cg20011352 | GeneID:25960 | *GPR124* | 8 | 495 | 0.000499 | 1.86 | CRC Hypermethylated | 0.215 | 27.492 | Novel |
| cg11102782 | GeneID:51477 | *ISYNA1* | 19 | 193 | 0.000122 | 2.80 | CRC Hypermethylated | 0.215 | 30.622 | Novel |
| cg00347904 | GeneID:222663 | *SCUBE3* | 6 | 318 | 6.33E-05 | 1.65 | CRC Hypermethylated | 0.215 | 29.958 | Novel |
| cg13547644 | GeneID:58 | *ACTA1* | 1 | 235 | 2.99E-05 | 1.55 | CRC Hypermethylated | 0.213 | 27.942 | Novel |
| cg18661868 | GeneID:2242 | *FES* | 15 | 256 | 5.78E-05 | 1.91 | CRC Hypermethylated | 0.213 | 34.892 | Novel |
| cg15747595 | GeneID:85453 | *TSPYL5* | 8 | 296 | 6.97E-05 | 1.45 | CRC Hypermethylated | 0.212 | 30.642 | Novel |
| cg18011401 | GeneID:773 | *CACNA1A* | 19 | 49 | 0.00057 | 2.83 | CRC Hypermethylated | 0.211 | 26.010 | Novel |
| cg03975694 | GeneID:163255 | *ZNF540* | 19 | 164 | 0.000117 | 1.46 | CRC Hypermethylated | 0.211 | 25.440 | Novel |
| cg16977035 | GeneID:60529 | *ALX4* | 11 | 0 | 3.99E-06 | 1.46 | CRC Hypermethylated | 0.211 | 37.233 | Previously reported |
| cg20279283 | GeneID:2626 | *GATA4* | 8 | 0 | 4.58E-07 | 1.41 | CRC Hypermethylated | 0.210 | 40.494 | Previously reported |
| cg00662556 | GeneID:2587 | *GALR1* | 18 | 0 | 3.46E-06 | 1.52 | CRC Hypermethylated | 0.210 | 37.039 | Previously reported |
| cg08441170 | GeneID:53904 | *MYO3A* | 10 | 96 | 5.01E-05 | 1.68 | CRC Hypermethylated | 0.209 | 25.058 | Novel |
| cg09573795 | GeneID:4487 | *MSX1* | 4 | 0 | 1.30E-07 | 1.46 | CRC Hypermethylated | 0.209 | 46.061 | Previously reported |
| cg21233722 | GeneID:1794 | *DOCK2* | 5 | 367 | 2.74E-07 | 1.46 | CRC Hypermethylated | 0.209 | 49.432 | Novel |
| cg17009433 | GeneID:2731 | *GLDC* | 9 | 36 | 0.000984 | 5.33 | CRC Hypermethylated | 0.208 | 21.609 | Novel |
| cg15105987 | GeneID:6657 | *SOX2* | 3 | 595 | 3.79E-07 | 1.59 | CRC Hypermethylated | 0.207 | 47.860 | Novel |
| cg19764418 | GeneID:6262 | *RYR2* | 1 | 604 | 8.34E-06 | 1.37 | CRC Hypermethylated | 0.206 | 45.133 | Novel |
| cg09748975 | GeneID:4487 | *MSX1* | 4 | 0 | 3.93E-09 | 1.29 | CRC Hypermethylated | 0.204 | 59.630 | Previously reported |
| cg21969640 | GeneID:53831 | *GPR84* | 12 | 681 | 9.66E-05 | -1.48 | CRC Hypomethylated | -0.208 | 31.306 | Novel |
| cg00750606 | GeneID:978 | *CDA* | 1 | 5 | 5.37E-05 | -1.58 | CRC Hypomethylated | -0.209 | 30.402 | Novel |
| cg15302379 | GeneID:81621 | *KAZALD1* | 10 | 250 | 2.82E-05 | -1.34 | CRC Hypomethylated | -0.211 | 37.528 | Novel |
| cg25538571 | GeneID:401459 | *FLJ46365* | 8 | 251 | 6.10E-05 | -1.61 | CRC Hypomethylated | -0.213 | 36.808 | Novel |
| cg20305726 | GeneID:81623 | *DEFB126* | 20 | 361 | 2.40E-05 | -1.66 | CRC Hypomethylated | -0.214 | 35.468 | Novel |
| cg17298704 | GeneID:51208 | *CLDN18* | 3 | 51 | 0.000183 | -1.30 | CRC Hypomethylated | -0.214 | 28.891 | Novel |
| cg21065959 | GeneID:353135 | *LCE1E* | 1 | 2 | 4.18E-07 | -1.37 | CRC Hypomethylated | -0.216 | 45.948 | Novel |
| cg21291985 | GeneID:140685 | *BTBD4* | 20 | 179 | 7.10E-06 | -1.56 | CRC Hypomethylated | -0.217 | 39.903 | Novel |
| cg07443748 | GeneID:150160 | *CESK1* | 22 | 106 | 1.21E-05 | -1.56 | CRC Hypomethylated | -0.218 | 35.410 | Novel |
| cg25214346 | GeneID:9970 | *NR1I3* | 1 | 11 | 8.99E-06 | -1.40 | CRC Hypomethylated | -0.218 | 37.014 | Novel |
| cg15669228 | GeneID:3445 | *IFNA8* | 9 | 372 | 3.70E-05 | -1.47 | CRC Hypomethylated | -0.219 | 33.066 | Novel |
| cg20556988 | GeneID:6346 | *CCL1* | 17 | 125 | 8.19E-05 | -1.39 | CRC Hypomethylated | -0.219 | 31.201 | Novel |
| cg24387380 | GeneID:2558 | *GABRA5* | 15 | 792 | 1.93E-07 | -1.80 | CRC Hypomethylated | -0.219 | 47.820 | Novel |
| cg17274742 | GeneID:10457 | *GPNMB* | 7 | 223 | 5.93E-07 | -1.50 | CRC Hypomethylated | -0.219 | 39.827 | Novel |
| cg08684473 | GeneID:10990 | *LILRB5* | 19 | 35 | 4.29E-05 | -1.35 | CRC Hypomethylated | -0.220 | 35.884 | Novel |
| cg15062535 | GeneID:162963 | *ZNF610* | 19 | 749 | 0.000254 | -1.43 | CRC Hypomethylated | -0.221 | 26.858 | Novel |
| cg26771272 | GeneID:125170 | *SMCR7* | 17 | 686 | 1.21E-08 | -1.52 | CRC Hypomethylated | -0.221 | 41.649 | Novel |
| cg13797031 | GeneID:8508 | *NIPSNAP1* | 22 | 414 | 3.42E-07 | -1.60 | CRC Hypomethylated | -0.222 | 41.847 | Novel |
| cg18833140 | GeneID:3026 | *HABP2* | 10 | 232 | 9.37E-07 | -1.58 | CRC Hypomethylated | -0.222 | 49.173 | Novel |
| cg05248470 | GeneID:10288 | *LILRB2* | 19 | 398 | 8.63E-06 | -1.58 | CRC Hypomethylated | -0.223 | 39.928 | Novel |
| cg20249919 | GeneID:5046 | *PCSK6* | 15 | 481 | 0.000245 | -1.37 | CRC Hypomethylated | -0.224 | 27.534 | Novel |
| cg11759378 | GeneID:151871 | *DPPA2* | 3 | 105 | 1.13E-06 | -1.43 | CRC Hypomethylated | -0.225 | 42.534 | Novel |
| cg14062083 | GeneID:284827 | *KRTAP13-4* | 21 | 235 | 9.14E-06 | -1.37 | CRC Hypomethylated | -0.225 | 36.194 | Novel |
| cg09120035 | GeneID:1584 | *CYP11B1* | 8 | 91 | 6.75E-05 | -1.37 | CRC Hypomethylated | -0.226 | 34.476 | Novel |
| cg03490200 | GeneID:144568 | *A2ML1* | 12 | 21 | 1.63E-06 | -1.39 | CRC Hypomethylated | -0.227 | 46.993 | Novel |
| cg02989940 | GeneID:51327 | *ERAF* | 16 | 31 | 3.13E-06 | -1.61 | CRC Hypomethylated | -0.227 | 37.561 | Novel |
| cg01615704 | GeneID:7851 | *MALL* | 2 | 252 | 1.10E-06 | -1.82 | CRC Hypomethylated | -0.227 | 46.177 | Novel |
| cg10057218 | GeneID:55876 | *GSDML* | 17 | 356 | 1.60E-06 | -1.47 | CRC Hypomethylated | -0.228 | 42.320 | Novel |
| cg19690214 | GeneID:26034 | *PIP3-E* | 6 | 426 | 9.61E-06 | -1.56 | CRC Hypomethylated | -0.229 | 39.656 | Novel |
| cg12331389 | GeneID:5950 | *RBP4* | 10 | 257 | 5.16E-07 | -2.66 | CRC Hypomethylated | -0.230 | 50.499 | Novel |
| cg22202141 | GeneID:2214 | *FCGR3A* | 1 | 773 | 3.86E-06 | -1.60 | CRC Hypomethylated | -0.230 | 36.710 | Novel |
| cg22268164 | GeneID:7201 | *TRHR* | 8 | 26 | 0.000558 | -1.52 | CRC Hypomethylated | -0.230 | 27.484 | Novel |
| cg05187322 | GeneID:79092 | *CARD14* | 17 | 47 | 4.16E-08 | -1.47 | CRC Hypomethylated | -0.231 | 40.404 | Novel |
| cg14603345 | GeneID:22903 | *BTBD3* | 20 | 102 | 4.99E-06 | -1.67 | CRC Hypomethylated | -0.231 | 39.616 | Novel |
| cg07084709 | GeneID:145781 | *Gcom1* | 15 | 603 | 2.61E-05 | -1.33 | CRC Hypomethylated | -0.231 | 37.830 | Novel |
| cg06255227 | GeneID:2832 | *NPBWR2* | 20 | 59 | 1.26E-05 | -1.36 | CRC Hypomethylated | -0.231 | 40.298 | Novel |
| cg11884243 | GeneID:2220 | *FCN2* | 9 | 228 | 2.87E-05 | -1.35 | CRC Hypomethylated | -0.231 | 34.555 | Novel |
| cg13396068 | GeneID:117159 | *DCD* | 12 | 767 | 9.49E-06 | -1.61 | CRC Hypomethylated | -0.231 | 40.679 | Novel |
| cg15520279 | GeneID:3234 | *HOXD8* | 2 | 611 | 1.48E-06 | -2.85 | CRC Hypomethylated | -0.231 | 52.013 | Novel |
| cg24101359 | GeneID:27232 | *GNMT* | 6 | 5 | 1.64E-05 | -2.67 | CRC Hypomethylated | -0.232 | 40.217 | Novel |
| cg24338843 | GeneID:93190 | *C1orf158* | 1 | 504 | 3.74E-06 | -1.54 | CRC Hypomethylated | -0.232 | 42.086 | Novel |
| cg23413307 | GeneID:353137 | *LCE1F* | 1 | 1376 | 0.000584 | -1.54 | CRC Hypomethylated | -0.233 | 25.279 | Novel |
| cg01668126 | GeneID:4481 | *MSR1* | 8 | 516 | 1.66E-06 | -1.48 | CRC Hypomethylated | -0.233 | 44.125 | Novel |
| cg08958913 | GeneID:56891 | *LGALS14* | 19 | 1056 | 5.65E-08 | -1.44 | CRC Hypomethylated | -0.233 | 35.320 | Novel |
| cg10345936 | GeneID:153201 | *SLC36A2* | 5 | 693 | 8.23E-07 | -1.78 | CRC Hypomethylated | -0.234 | 44.637 | Novel |
| cg01055695 | GeneID:5047 | *PAEP* | 9 | 654 | 3.78E-05 | -1.44 | CRC Hypomethylated | -0.234 | 33.897 | Novel |
| cg24244000 | GeneID:2567 | *GABRG3* | 15 | 165 | 6.55E-06 | -1.41 | CRC Hypomethylated | -0.235 | 41.378 | Novel |
| cg10533434 | GeneID:6317 | *SERPINB3* | 18 | 112 | 3.56E-07 | -1.62 | CRC Hypomethylated | -0.235 | 51.758 | Novel |
| cg07879977 | GeneID:4992 | *OR1F1* | 16 | 320 | 5.18E-06 | -1.56 | CRC Hypomethylated | -0.236 | 37.026 | Novel |
| cg00614413 | GeneID:5802 | *PTPRS* | 19 | 195 | 2.87E-06 | -1.60 | CRC Hypomethylated | -0.236 | 38.244 | Novel |
| cg18992201 | GeneID:151871 | *DPPA2* | 3 | 17 | 2.88E-07 | -1.41 | CRC Hypomethylated | -0.236 | 48.557 | Novel |
| cg14934821 | GeneID:26086 | *GPSM1* | 9 | 85 | 4.65E-05 | -1.52 | CRC Hypomethylated | -0.236 | 29.114 | Novel |
| cg17628717 | GeneID:23072 | *HECW1* | 7 | 154 | 2.36E-06 | -1.52 | CRC Hypomethylated | -0.237 | 43.350 | Novel |
| cg16483466 | GeneID:149954 | *C20orf186* | 20 | 479 | 1.55E-05 | -1.45 | CRC Hypomethylated | -0.237 | 40.963 | Novel |
| cg25406518 | GeneID:26007 | *DAK* | 11 | 15 | 2.14E-07 | -1.69 | CRC Hypomethylated | -0.238 | 46.072 | Novel |
| cg02121427 | GeneID:131578 | *LRRC15* | 3 | 720 | 1.64E-08 | -2.08 | CRC Hypomethylated | -0.238 | 40.364 | Novel |
| cg21505886 | GeneID:92305 | *TMEM129* | 4 | 1344 | 7.52E-07 | -1.56 | CRC Hypomethylated | -0.238 | 42.813 | Novel |
| cg27000831 | GeneID:6355 | *CCL8* | 17 | 318 | 5.05E-08 | -1.74 | CRC Hypomethylated | -0.238 | 51.677 | Novel |
| cg00474004 | GeneID:3448 | *IFNA14* | 9 | 46 | 1.56E-05 | -1.52 | CRC Hypomethylated | -0.239 | 38.863 | Novel |
| cg06793062 | GeneID:85445 | *CNTNAP4* | 16 | 119 | 1.21E-05 | -1.48 | CRC Hypomethylated | -0.239 | 42.914 | Novel |
| cg24776407 | GeneID:26579 | *MYEOV* | 11 | 12 | 4.91E-06 | -1.48 | CRC Hypomethylated | -0.239 | 43.898 | Novel |
| cg25612145 | GeneID:56164 | *STK31* | 7 | 252 | 4.12E-07 | -1.35 | CRC Hypomethylated | -0.240 | 52.876 | Novel |
| cg19391527 | GeneID:5368 | *PNOC* | 8 | 917 | 8.34E-07 | -1.84 | CRC Hypomethylated | -0.240 | 47.197 | Novel |
| cg25477904 | GeneID:5669 | *PSG1* | 19 | 466 | 6.35E-06 | -1.39 | CRC Hypomethylated | -0.240 | 40.540 | Novel |
| cg16879115 | GeneID:339 | *APOBEC1* | 12 | 678 | 9.81E-10 | -1.70 | CRC Hypomethylated | -0.241 | 51.246 | Novel |
| cg04574507 | GeneID:910 | *CD1B* | 1 | 220 | 2.03E-07 | -1.48 | CRC Hypomethylated | -0.241 | 48.389 | Novel |
| cg00134787 | GeneID:4619 | *MYH1* | 17 | 460 | 6.36E-06 | -1.43 | CRC Hypomethylated | -0.242 | 44.427 | Novel |
| cg06577725 | GeneID:83893 | *SPATA16* | 3 | 84 | 1.14E-05 | -1.54 | CRC Hypomethylated | -0.242 | 34.459 | Novel |
| cg14662172 | GeneID:1361 | *CPB2* | 13 | 87 | 0.000136 | -1.51 | CRC Hypomethylated | -0.242 | 32.467 | Novel |
| cg23248452 | GeneID:50834 | *TAS2R1* | 5 | 654 | 2.96E-06 | -1.60 | CRC Hypomethylated | -0.243 | 40.129 | Novel |
| cg15642035 | GeneID:51059 | *C8ORFK32* | 8 | 405 | 5.57E-05 | -1.61 | CRC Hypomethylated | -0.243 | 31.956 | Novel |
| cg16894211 | GeneID:8224 | *SYN3* | 22 | 859 | 1.54E-06 | -1.79 | CRC Hypomethylated | -0.243 | 42.189 | Novel |
| cg20311730 | GeneID:338322 | *NALP10* | 11 | 135 | 2.74E-05 | -1.39 | CRC Hypomethylated | -0.244 | 37.872 | Novel |
| cg14757492 | GeneID:54555 | *DDX49* | 19 | 1300 | 6.85E-09 | -1.52 | CRC Hypomethylated | -0.244 | 51.158 | Novel |
| cg12818699 | GeneID:9750 | *C6orf32* | 6 | 1 | 1.45E-05 | -1.43 | CRC Hypomethylated | -0.244 | 39.017 | Novel |
| cg22658979 | GeneID:4322 | *MMP13* | 11 | 7 | 3.91E-06 | -1.43 | CRC Hypomethylated | -0.245 | 42.445 | Novel |
| cg11435943 | GeneID:89777 | *SERPINB12* | 18 | 706 | 0.000201 | -1.58 | CRC Hypomethylated | -0.245 | 29.393 | Novel |
| cg11871280 | GeneID:9194 | *SLC16A7* | 12 | 1088 | 7.83E-06 | -1.48 | CRC Hypomethylated | -0.247 | 36.844 | Novel |
| cg25229305 | GeneID:338567 | *KCNK18* | 10 | 361 | 3.59E-06 | -1.78 | CRC Hypomethylated | -0.247 | 42.064 | Novel |
| cg14826683 | GeneID:6703 | *SPRR2D* | 1 | 574 | 1.18E-06 | -1.71 | CRC Hypomethylated | -0.248 | 45.189 | Novel |
| cg24870391 | GeneID:6356 | *CCL11* | 17 | 12 | 1.76E-06 | -1.57 | CRC Hypomethylated | -0.248 | 46.821 | Novel |
| cg26164184 | GeneID:2220 | *FCN2* | 9 | 1152 | 1.10E-05 | -1.47 | CRC Hypomethylated | -0.250 | 36.593 | Novel |
| cg12493906 | GeneID:56547 | *MMP26* | 11 | 113 | 1.08E-05 | -1.64 | CRC Hypomethylated | -0.250 | 35.565 | Novel |
| cg10054857 | GeneID:221241 | *C18orf20* | 18 | 279 | 2.19E-06 | -1.69 | CRC Hypomethylated | -0.251 | 44.078 | Novel |
| cg08458487 | GeneID:6441 | *SFTPD* | 10 | 330 | 2.05E-06 | -1.52 | CRC Hypomethylated | -0.252 | 44.710 | Novel |
| cg26111757 | GeneID:359710 | *C20orf185* | 20 | 1110 | 1.34E-06 | -1.72 | CRC Hypomethylated | -0.252 | 43.301 | Novel |
| cg09440243 | GeneID:5789 | *PTPRD* | 9 | 1022 | 3.50E-05 | -1.47 | CRC Hypomethylated | -0.253 | 36.528 | Novel |
| cg23110514 | GeneID:353145 | *LCE3E* | 1 | 360 | 1.40E-07 | -1.82 | CRC Hypomethylated | -0.254 | 40.364 | Novel |
| cg10305797 | GeneID:388533 | *UNQ467* | 19 | 321 | 8.56E-08 | -1.74 | CRC Hypomethylated | -0.254 | 51.709 | Novel |
| cg07711097 | GeneID:2765 | *GML* | 8 | 263 | 8.26E-06 | -1.41 | CRC Hypomethylated | -0.255 | 40.175 | Previously reported |
| cg13944141 | GeneID:5645 | *PRSS2* | 7 | 46 | 1.76E-06 | -1.72 | CRC Hypomethylated | -0.255 | 43.870 | Novel |
| cg19297688 | GeneID:3641 | *INSL4* | 9 | 59 | 9.64E-07 | -1.63 | CRC Hypomethylated | -0.255 | 34.912 | Novel |
| cg02347487 | GeneID:338323 | *NALP14* | 11 | 694 | 3.92E-08 | -2.11 | CRC Hypomethylated | -0.256 | 52.768 | Novel |
| cg21491308 | GeneID:83639 | *TEX101* | 19 | 63 | 2.47E-06 | -1.65 | CRC Hypomethylated | -0.257 | 44.088 | Novel |
| cg22809047 | GeneID:6160 | *RPL31* | 2 | 490 | 0.00014 | -1.51 | CRC Hypomethylated | -0.257 | 34.194 | Novel |
| cg04282622 | GeneID:196472 | *FAM71C* | 12 | 609 | 6.99E-07 | -1.83 | CRC Hypomethylated | -0.257 | 50.876 | Novel |
| cg11009736 | GeneID:8685 | *MARCO* | 2 | 63 | 2.39E-06 | -1.86 | CRC Hypomethylated | -0.258 | 46.159 | Novel |
| cg01309152 | GeneID:5121 | *PCP4* | 21 | 424 | 1.78E-06 | -1.41 | CRC Hypomethylated | -0.258 | 43.198 | Novel |
| cg14547335 | GeneID:491 | *ATP2B2* | 3 | 299 | 2.36E-05 | -1.40 | CRC Hypomethylated | -0.258 | 37.873 | Novel |
| cg06353345 | GeneID:79339 | *OR51B4* | 11 | 200 | 8.58E-07 | -1.91 | CRC Hypomethylated | -0.259 | 48.969 | Novel |
| cg14462830 | GeneID:10345 | *TRDN* | 6 | 1242 | 6.95E-06 | -1.63 | CRC Hypomethylated | -0.259 | 40.212 | Novel |
| cg20383064 | GeneID:8419 | *BFSP2* | 3 | 1370 | 5.60E-05 | -1.48 | CRC Hypomethylated | -0.259 | 34.223 | Novel |
| cg09906458 | GeneID:23627 | *PRND* | 20 | 25 | 8.82E-06 | -1.61 | CRC Hypomethylated | -0.260 | 28.053 | Novel |
| cg04384208 | GeneID:2214 | *FCGR3A* | 1 | 422 | 2.24E-07 | -1.66 | CRC Hypomethylated | -0.260 | 35.510 | Novel |
| cg12878228 | GeneID:5644 | *PRSS1* | 7 | 1206 | 3.38E-06 | -1.66 | CRC Hypomethylated | -0.260 | 40.518 | Previously reported |
| cg04567009 | GeneID:2215 | *FCGR3B* | 1 | 132 | 2.14E-06 | -1.56 | CRC Hypomethylated | -0.260 | 40.751 | Novel |
| cg04329382 | GeneID:400931 | *FLJ27365* | 22 | 992 | 1.75E-07 | -1.64 | CRC Hypomethylated | -0.260 | 47.395 | Novel |
| cg20676475 | GeneID:84648 | *LCE3D* | 1 | 486 | 9.04E-07 | -1.63 | CRC Hypomethylated | -0.261 | 45.424 | Novel |
| cg21094154 | GeneID:8600 | *TNFSF11* | 13 | 326 | 1.39E-09 | -1.89 | CRC Hypomethylated | -0.261 | 50.233 | Novel |
| cg01671881 | GeneID:128861 | *C20orf71* | 20 | 257 | 2.11E-05 | -1.74 | CRC Hypomethylated | -0.262 | 37.954 | Novel |
| cg14696870 | GeneID:2205 | *FCER1A* | 1 | 627 | 7.03E-06 | -1.50 | CRC Hypomethylated | -0.265 | 40.788 | Novel |
| cg07373172 | GeneID:3452 | *IFNA21* | 9 | 374 | 3.24E-06 | -1.54 | CRC Hypomethylated | -0.266 | 47.091 | Novel |
| cg03109316 | GeneID:7634 | *ZNF80* | 3 | 1379 | 8.23E-06 | -1.46 | CRC Hypomethylated | -0.267 | 39.750 | Novel |
| cg10503138 | GeneID:152330 | *CNTN4* | 3 | 144 | 5.58E-07 | -1.59 | CRC Hypomethylated | -0.268 | 49.176 | Novel |
| cg01772980 | GeneID:10648 | *SCGB1D1* | 11 | 15 | 2.34E-07 | -1.58 | CRC Hypomethylated | -0.268 | 45.544 | Novel |
| cg14338887 | GeneID:27232 | *GNMT* | 6 | 0 | 4.21E-08 | -2.52 | CRC Hypomethylated | -0.270 | 59.472 | Novel |
| cg26738880 | GeneID:1804 | *DPP6* | 7 | 499 | 5.52E-06 | -1.81 | CRC Hypomethylated | -0.270 | 37.358 | Novel |
| cg18223379 | GeneID:128859 | *BPIL3* | 20 | 193 | 2.35E-06 | -1.65 | CRC Hypomethylated | -0.271 | 41.758 | Novel |
| cg05241571 | GeneID:388533 | *UNQ467* | 19 | 132 | 6.29E-06 | -1.44 | CRC Hypomethylated | -0.272 | 41.526 | Novel |
| cg14898779 | GeneID:56164 | *STK31* | 7 | 284 | 1.18E-06 | -1.48 | CRC Hypomethylated | -0.273 | 50.228 | Novel |
| cg14893129 | GeneID:79092 | *CARD14* | 17 | 230 | 7.00E-08 | -1.53 | CRC Hypomethylated | -0.275 | 56.477 | Novel |
| cg03116740 | GeneID:7106 | *TSPAN4* | 11 | 1489 | 4.02E-07 | -1.50 | CRC Hypomethylated | -0.276 | 48.193 | Novel |
| cg10159529 | GeneID:3568 | *IL5RA* | 3 | 499 | 4.26E-09 | -1.59 | CRC Hypomethylated | -0.281 | 60.066 | Novel |
| cg07525077 | GeneID:6037 | *RNASE3* | 14 | 381 | 1.99E-06 | -1.70 | CRC Hypomethylated | -0.281 | 45.810 | Novel |
| cg14894216 | GeneID:117144 | *CATSPER1* | 11 | 368 | 8.05E-08 | -1.67 | CRC Hypomethylated | -0.283 | 49.190 | Novel |
| cg15051063 | GeneID:5088 | *PBX2P1* | 3 | 957 | 5.20E-07 | -1.52 | CRC Hypomethylated | -0.283 | 50.537 | Novel |
| cg08878744 | GeneID:353132 | *LCE1B* | 1 | 773 | 1.82E-06 | -1.87 | CRC Hypomethylated | -0.284 | 47.433 | Novel |
| cg04893119 | GeneID:51050 | *PI15* | 8 | 471 | 9.30E-08 | -1.79 | CRC Hypomethylated | -0.285 | 41.260 | Novel |
| cg27043873 | GeneID:1016 | *CDH18* | 5 | 140 | 1.13E-05 | -1.67 | CRC Hypomethylated | -0.286 | 41.894 | Novel |
| cg02656594 | GeneID:50615 | *IL21R* | 16 | 1227 | 1.11E-11 | -2.60 | CRC Hypomethylated | -0.286 | 71.987 | Novel |
| cg21312148 | GeneID:353141 | *LCE2D* | 1 | 202 | 1.34E-07 | -1.70 | CRC Hypomethylated | -0.289 | 48.241 | Novel |
| cg18678121 | GeneID:55176 | *SEC61A2* | 10 | 891 | 1.21E-06 | -1.57 | CRC Hypomethylated | -0.294 | 39.870 | Novel |
| cg26390526 | GeneID:2312 | *FLG* | 1 | 1399 | 6.24E-06 | -1.58 | CRC Hypomethylated | -0.294 | 39.317 | Novel |
| cg14940420 | GeneID:389123 | *IQCF2* | 3 | 47 | 3.92E-06 | -1.66 | CRC Hypomethylated | -0.297 | 45.167 | Novel |
| cg20399252 | GeneID:84650 | *EBPL* | 13 | 616 | 2.19E-07 | -1.64 | CRC Hypomethylated | -0.301 | 42.595 | Novel |
| cg13696012 | GeneID:80341 | *BPIL1* | 20 | 47 | 2.71E-09 | -1.58 | CRC Hypomethylated | -0.301 | 59.499 | Novel |
| cg27513764 | GeneID:146779 | *EFCAB3* | 17 | 47 | 1.57E-07 | -1.68 | CRC Hypomethylated | -0.302 | 50.872 | Novel |
| cg11639651 | GeneID:80201 | *HKDC1* | 10 | 248 | 6.54E-07 | -1.60 | CRC Hypomethylated | -0.309 | 51.238 | Novel |
| cg08696192 | GeneID:84951 | *TNS4* | 17 | 505 | 4.16E-08 | -1.55 | CRC Hypomethylated | -0.309 | 56.816 | Novel |
| cg27032184 | GeneID:55687 | *TRMU* | 22 | 193 | 5.41E-06 | -1.90 | CRC Hypomethylated | -0.317 | 45.925 | Novel |
| cg05445326 | GeneID:116211 | *TM4SF19* | 3 | 325 | 3.06E-08 | -1.58 | CRC Hypomethylated | -0.319 | 53.814 | Novel |
| cg18462653 | GeneID:245932 | *DEFB119* | 20 | 230 | 1.53E-06 | -1.91 | CRC Hypomethylated | -0.319 | 44.728 | Novel |
| cg07456201 | GeneID:7369 | *UMOD* | 16 | 326 | 6.75E-08 | -2.02 | CRC Hypomethylated | -0.325 | 54.241 | Novel |
| cg11762346 | GeneID:80201 | *HKDC1* | 10 | 53 | 9.05E-07 | -1.55 | CRC Hypomethylated | -0.325 | 50.330 | Novel |
| cg22266967 | GeneID:6286 | *S100P* | 4 | 132 | 5.89E-08 | -1.90 | CRC Hypomethylated | -0.331 | 53.543 | Novel |
| cg10968815 | GeneID:80341 | *BPIL1* | 20 | 61 | 1.39E-08 | -1.97 | CRC Hypomethylated | -0.350 | 62.061 | Novel |
| cg12958813 | GeneID:127124 | *ATP6V1G3* | 1 | 406 | 8.31E-08 | -1.81 | CRC Hypomethylated | -0.353 | 53.657 | Novel |
| cg02442161 | GeneID:5266 | *PI3* | 20 | 139 | 1.79E-10 | -2.16 | CRC Hypomethylated | -0.412 | 66.452 | Previously reported |
| The loci are sorted by Delta Beta (magnitude of differential methylation) in descending order. CHR: chromosome; TSS: transcription start site; *ANOVA p-value adjusted for sex & location of the tumor; Delta Beta: Beta value of CRC minus beta value of normal; **PC_tissue: variability of the beta value that can be explained by type of tissue (CRC or normal); For "previously reported markers", please see Kim et al [27] and Ang et al.[9] | | | | | | | | | | |
|

| **Additional File 1 Table S4**  **Validation of microarray methylation data by qPCR-based methyl profiler assay of twelve genes in paired samples from 10 patients (20 samples).** | | | | | | | | |
| --- | --- | --- | --- | --- | --- | --- | --- | --- |
|  | | N | Mean proportion of methylation* in total DNA by q-PCR | 95% Confidence Interval for Mean | | ANOVA-4way** | | Correlation (r=) of q-PCR methylation with microarray Beta |
|  |  |  |  | Lower Bound | Upper Bound | F | p-value |  |
| *TRH* | Normal | 10 | 0.0312 | 0.0146 | 0.0477 | 93.08 | 4.82E-06 | 0.810590 |
|  | CRC | 10 | 0.6322 | 0.4943 | 0.7702 |  |  |  |
| *C2orf32* | Normal | 10 | 0.0063 | 0.0044 | 0.0082 | 74.80 | 1.18E-05 | 0.928307 |
|  | CRC | 10 | 0.5971 | 0.4430 | 0.7513 |  |  |  |
| *FLJ25477* | Normal | 10 | 0.1365 | -0.0232 | 0.2962 | 48.41 | 6.63E-05 | 0.928536 |
|  | CRC | 10 | 0.7247 | 0.6566 | 0.7929 |  |  |  |
| *ITGA4* | Normal | 10 | 0.0032 | 0.0022 | 0.0041 | 38.23 | 1.62E-04 | 0.941483 |
|  | CRC | 10 | 0.5023 | 0.3196 | 0.6850 |  |  |  |
| *KCNQ5* | Normal | 10 | 0.0144 | 0.0011 | 0.0277 | 31.45 | 3.31E-04 | 0.597506 |
|  | CRC | 10 | 0.4534 | 0.2806 | 0.6262 |  |  |  |
| *C1orf165* | Normal | 10 | 0.0021 | 0.0014 | 0.0028 | 22.27 | 1.09E-03 | 0.914588 |
|  | CRC | 10 | 0.4001 | 0.2094 | 0.5909 |  |  |  |
| *FLI1* | Normal | 10 | 0.0054 | 0.0009 | 0.0099 | 17.01 | 2.58E-03 | 0.798248 |
|  | CRC | 10 | 0.4143 | 0.1901 | 0.6384 |  |  |  |
| *MDFI* | Normal | 10 | 0.0271 | 0.0193 | 0.0348 | 12.69 | 6.10E-03 | 0.710942 |
|  | CRC | 10 | 0.3862 | 0.1615 | 0.6109 |  |  |  |
| *DAB2IP* | Normal | 10 | 0.0320 | 0.0231 | 0.0409 | 12.54 | 6.30E-03 | 0.809820 |
|  | CRC | 10 | 0.2606 | 0.1154 | 0.4058 |  |  |  |
| *RIC3* | Normal | 10 | 0.3930 | 0.1734 | 0.6126 | 6.57 | 3.06E-02 | 0.523752 |
|  | CRC | 10 | 0.5835 | 0.4176 | 0.7493 |  |  |  |
| *RYR2* | Normal | 10 | 0.5930 | 0.4427 | 0.7432 | 2.86 | *1.25E-01* | *0.410544* |
|  | CRC | 10 | 0.7360 | 0.6667 | 0.8053 |  |  |  |
| *ZNF625* | Normal | 10 | 0.1014 | 0.0749 | 0.1279 | 2.63 | *1.40E-01* | *0.538650* |
|  | CRC | 10 | 0.1580 | 0.0814 | 0.2346 |  |  |  |
| Differential methylation in CRC tissue compared to corresponding normal tissue as well as the correlation of q-PCR data with microarray data (beta-value) is shown.  * Proportion of hypermethylated & intermidiate methylated DNA in q-PCR-based methyl profiler assay was combined to get proportion of methylated DNA. **In 4-way ANOVA, tissue type (CRC or normal) is adjusted for inter-person variation, sex and location of the tumor | | | | | | | | |
