# Supplementary figures and images for: A genome-wide DNA methylation study in colorectal carcinoma
Source: BMC Med Genomics. 2011 Jun 23;4:50. doi: 10.1186/1755-8794-4-50 (PMC3135506; doi:10.1186/1755-8794-4-50)

## Slide 1
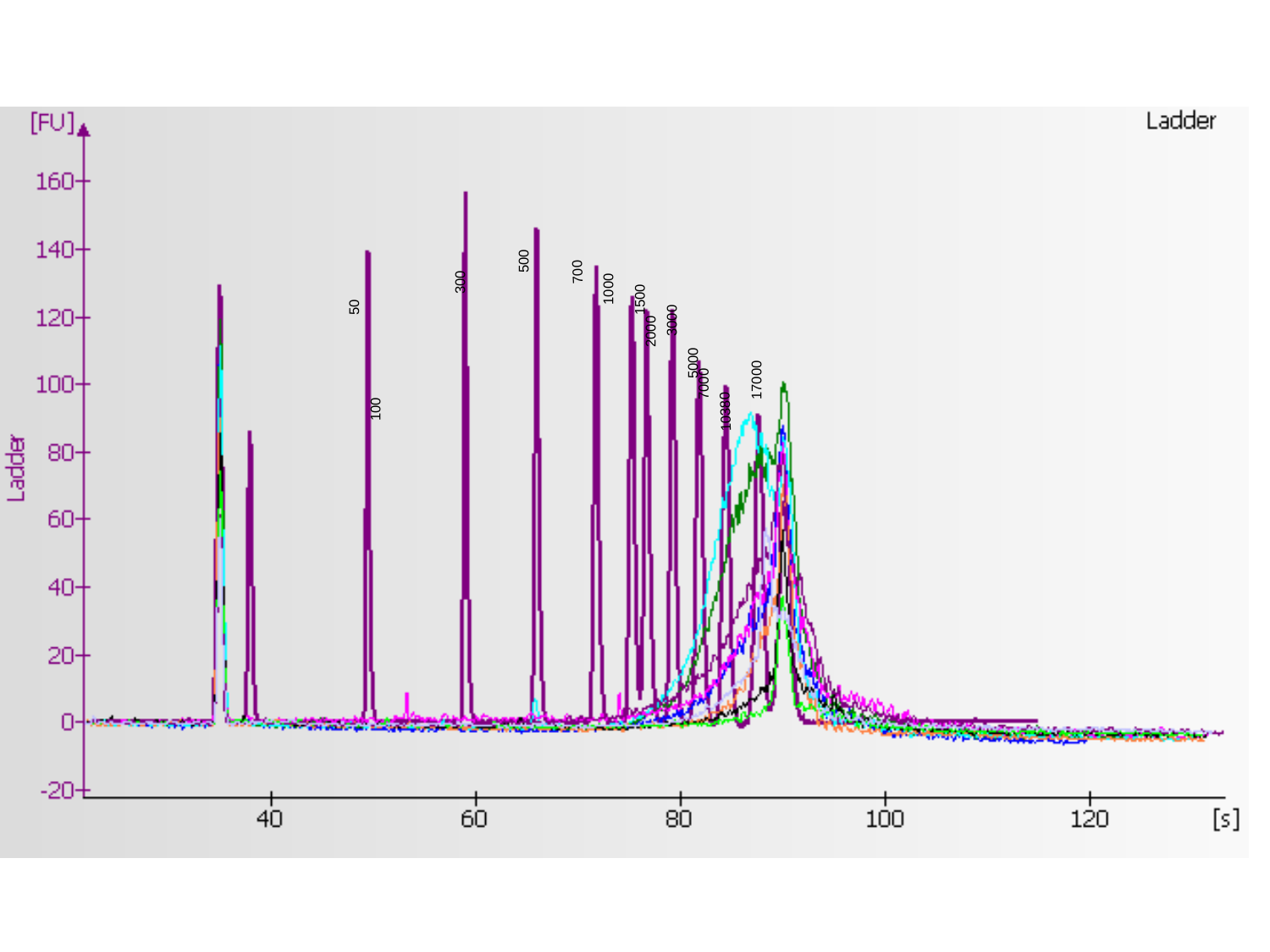

500
700
300
1000
50
1500
3000
2000
5000
7000
17000
100
10380

Supplement: Additional File 2 — Figure S1: Electropherogram of DNA samples. Agilent 2100 BioAnalyzer electropherogram of 10 DNA samples (in different colors) overlaid on ladder marker (shown in violet). Size (bp) of each peak of the DNA ladder in shown on the top of each peak. The figure shows DNA fragment size >10000 bp. [file 1755-8794-4-50-S2.PPT]

## Slide 1
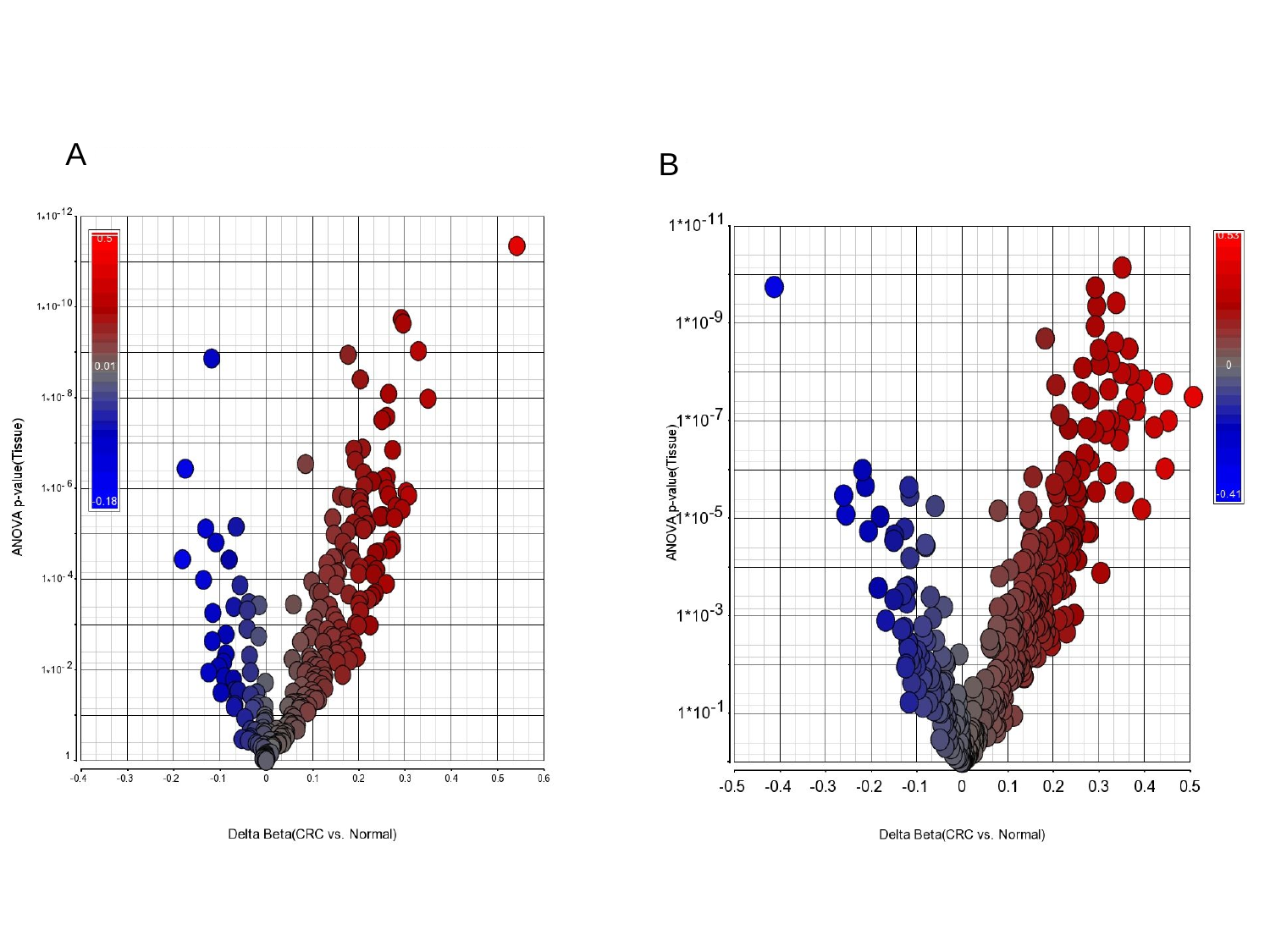

A
B

Supplement: Additional File 3 — Figure S2: Volcano plot showing methylation status of previously reported genes in our samples. The Delta β is shown on x-axis and ANOVA p-value on the y-axis. A: represents the 245 loci covering the previously reported genes mainly from candidate gene approach-based studies; B: represents the 376 loci covering the 132 genes reported from a single study based on genome-wide approach (although testing only 1505 CpG sites). The side bar shows the color scale depending on Delta β where blue indicates hypomethylation and red indicates hypermethylation in CRC. [file 1755-8794-4-50-S3.PPT]

## Slide 1
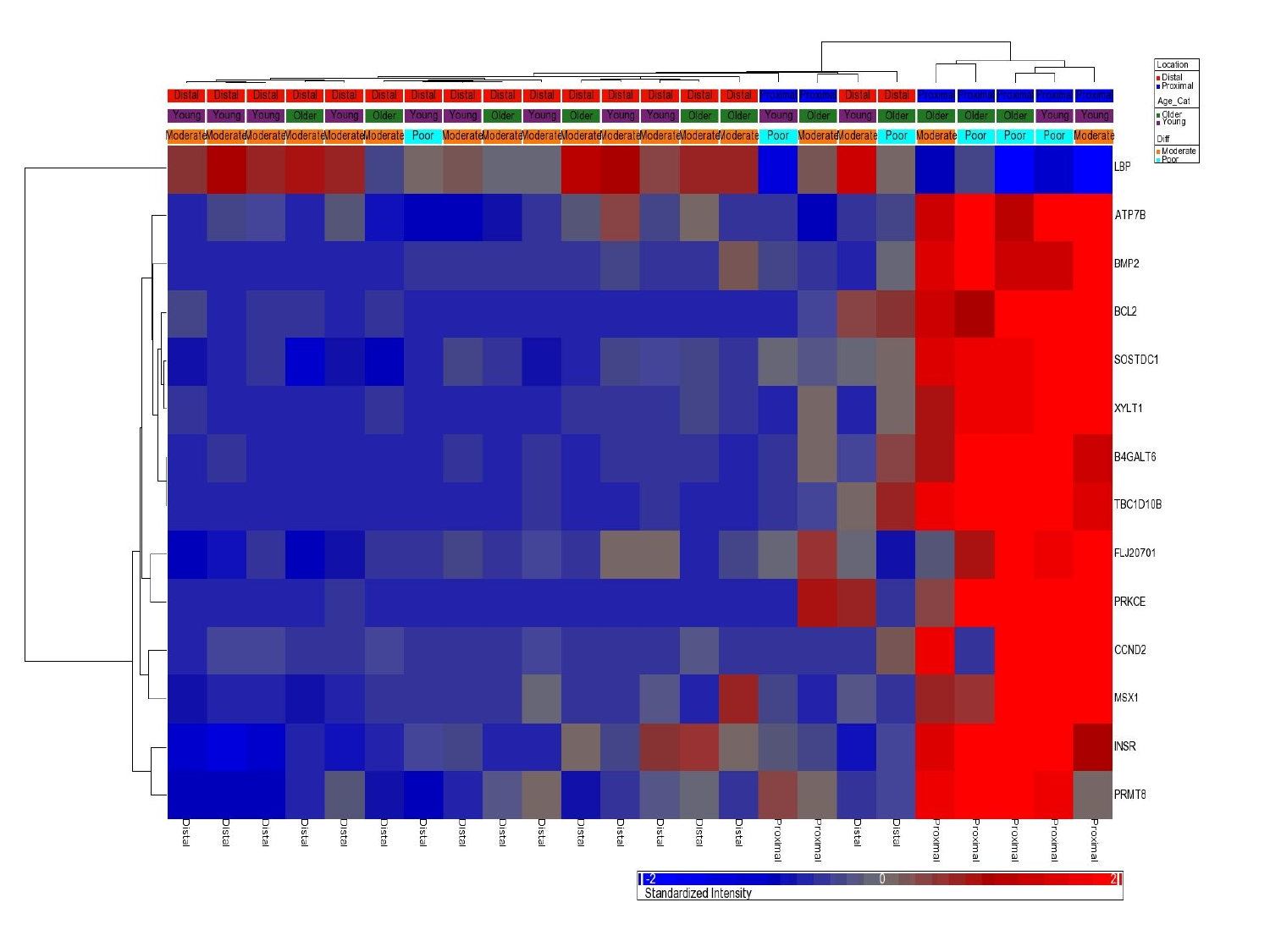

Supplement: Additional File 4 — Figure S3: Heatmap of 14 loci that are differentially methylated in proximal CRC compared to distal CRC. Unsupervised hierarchical clustering of the 14 loci (rows) in 24 CRC samples (columns). Thirteen of these loci were hypermethylated in proximal CRC compared to distal CRC. The two major clusters generated (top dendogram) in this analysis separated most of the proximal CRC tissues from the distal CRC tissues. Age at diagnosis (>45 yrs or = <45 yrs and the differentiation of the tumor (moderately differentiated or poorly differentiated) are shown above the heatmap. [file 1755-8794-4-50-S4.PPT]

## Slide 1
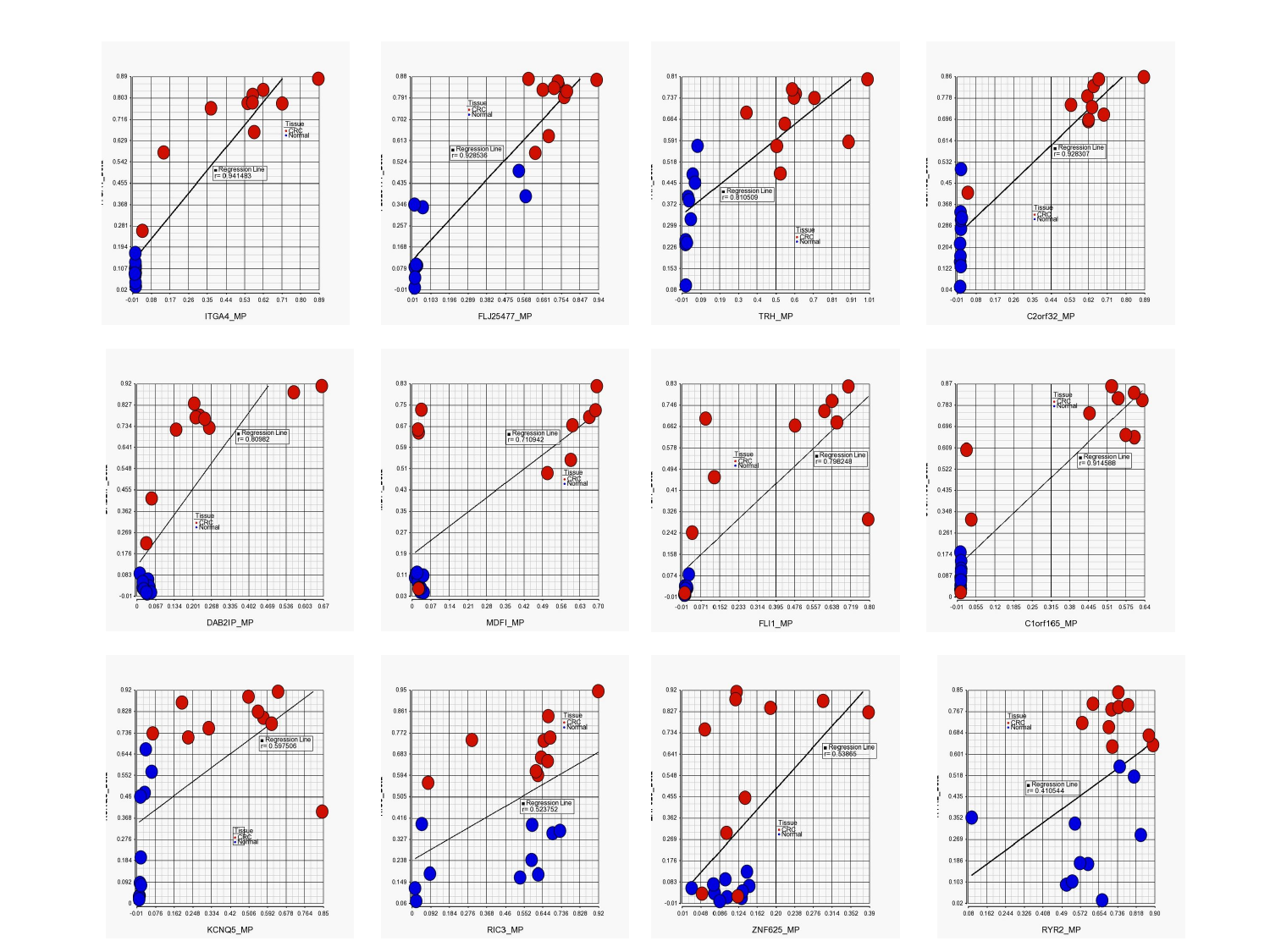

Supplement: Additional File 5 — Figure S4. Comparison between q-PCR and microarray methylation data. Graphs are shown for the 12 genes validated by q-PCR. The y-axis plots the β value from microarray data. The x-axis plots the proportion of intermediately methylated and hypermethylated DNA. [file 1755-8794-4-50-S5.PPT]
